# CD36/SR-B2-TLR2 Dependent Pathways Enhance *Porphyromonas gingivalis* Mediated Atherosclerosis in the *Ldlr* KO Mouse Model

**DOI:** 10.1371/journal.pone.0125126

**Published:** 2015-05-04

**Authors:** Paul M. Brown, David J. Kennedy, Richard E. Morton, Maria Febbraio

**Affiliations:** 1 Pediatric Research Center, Lerner Research Institute, Cleveland Clinic, Cleveland, Ohio, United States of America; 2 Department of Cellular and Molecular Medicine, Lerner Research Institute, Cleveland Clinic, Cleveland, Ohio, United States of America; 3 Department of Molecular Medicine, Cleveland Clinic Lerner College of Medicine of Case Western Reserve University, Cleveland, Ohio, United States of America; 4 Department of Dentistry, University of Alberta, Edmonton, Alberta, Canada; Massachusetts General Hospital and Harvard Medical School, UNITED STATES

## Abstract

There is strong epidemiological association between periodontal disease and cardiovascular disease but underlying mechanisms remain ill-defined. Because the human periodontal disease pathogen, *Porphyromonas gingivalis* (Pg), interacts with innate immune receptors Toll-like Receptor (TLR) 2 and CD36/scavenger receptor-B2 (SR-B2), we studied how CD36/SR-B2 and TLR pathways promote Pg-mediated atherosclerosis. Western diet fed low density lipoprotein receptor knockout (*Ldlr*°) mice infected orally with Pg had a significant increase in lesion burden compared with uninfected controls. This increase was entirely CD36/SR-B2-dependent, as there was no significant change in lesion burden between infected and uninfected *Ldlr*° mice. Western diet feeding promoted enhanced CD36/SR-B2-dependent IL1β generation and foam cell formation as a result of Pg lipopolysaccharide (PgLPS) exposure. CD36/SR-B2 and TLR2 were necessary for inflammasome activation and optimal IL1ß generation, but also resulted in LPS induced lethality (pyroptosis). Modified forms of LDL inhibited Pg-mediated IL1ß generation in a CD36/SR-B2-dependent manner and prevented pyroptosis, but promoted foam cell formation. Our data show that Pg infection in the oral cavity can lead to significant TLR2-CD36/SR-B2 dependent IL1ß release. In the vessel wall, macrophages encountering systemic release of IL1ß, PgLPS and modified LDL have increased lipid uptake, foam cell formation, and release of IL1ß, but because pyroptosis is inhibited, this enables macrophage survival and promotes increased plaque development. These studies may explain increased lesion burden as a result of periodontal disease, and suggest strategies for development of therapeutics.

## Introduction

In the US, greater than 20% of adults aged 35–64 have moderate to severe periodontal disease (PD), and this is likely underestimated [1–3]. Oral pathogens, including *Porphyromonas gingivalis* (Pg), contribute to PD directly and indirectly by activating a destructive immune response and by altering the environmental milieu and microbiome [4–7]. Epidemiological and experimental research support an association between PD and cardiovascular disease, but the mechanism(s) underlying this association are not fully understood [8–12]. An important question has emerged: how is a localized and largely isolated oral infection transduced to the aortic vasculature to promote atherosclerosis? While there has been substantive progress in reducing risk associated with dyslipidemia in treatment of atherosclerosis, there has been less therapeutic focus on chronic infectious diseases such as PD [13]. The prevalence of PD, its association with diabetes and obesity, which are increasing rapidly globally, and its increased severity in the elderly population, underscore the necessity for research in this area and the significance of the problem [14–17].

Previous work has indicated that Toll-Like Receptors (TLRs) 2 and 4 have complex roles in infections with Pg [18–24]. While TLR2 has been shown to be pro-atherogenic in a mouse model of PD and atherosclerosis, TLR4 reduces lesion burden [25–29]. The macrophage scavenger receptor CD36/SR-B2 is also an important mediator of atherosclerosis, through recognition and internalization of modified pro-atherogenic LDL leading to foam cell formation and a signaling cascade that further promotes inflammation at the vessel wall via secretion of cytokines and ROS [30–34]. CD36/SR-B2 co-operates with TLRs in several responses, including acting as a co-receptor with TLR2 for gram positive bacteria [35–39]. Given the overlap in interactions amongst CD36/SR-B2, TLRs and Pg, we hypothesized that there may be a CD36/SR-B2-dependent aspect to the mechanism of PD associated atherosclerosis. Using Western diet (WD) fed *Ldlr*° mice orally infected with Pg, we found a significant 225% (females) and 175% (males) increase in lesion burden compared with uninfected controls. This increase was entirely CD36/SR-B2-dependent, as there was no significant change in lesion burden between infected and uninfected *Cd36*°*/Ldlr*° mice. These data introduce CD36/SR-B2 as an essential receptor involved in the link between Pg and cardiovascular disease.

Mechanistically, we show that Pg-mediated enhancment of foam cell formation is dependent on CD36/SR-B2 and that both CD36/SR-B2 and TLR2 are necessary for optimal IL1β generation. Modified LDL and the specific CD36/SR-B2 ligand, 1-(Palmitoyl)-2-(5-keto-6-octene-dioyl) phosphatidylcholine (KOdiA-PC), inhibited Pg-mediated IL1β generation and pyroptosis. We propose a model in which macrophages of the oral cavity, upon encounter with Pg, activate the inflammasome in a CD36/SR-B2-TLR2 dependent manner resulting in systemic release of IL1β. In the vessel wall, naïve macrophage stimulation with systemic IL1β leads to localized IL1β release, enhancement of lipid uptake, foam cell formation and increased lesion burden, but as a result of a high fat, Western style diet and modified LDL, pyroptosis is prevented in response to Pg. Systemic IL1β as a result of oral cavity Pg infection, in association with Pg-mediated enhanced CD36/SR-B2-oxidized LDL (oxLDL) macrophage lipid uptake and inhibition of pyroptosis in the vasculature, may be key to enhanced atherosclerosis in PD and present novel potential targets for development of treatment strategies.

## Materials and Methods

### Reagents

Please see S1 File, Reagent List for a detailed list of reagents.

### Mice

All animal procedures were prior approved by the Institutional Animal Care and Use Committee of Cleveland Clinic and carried out in an AAALAC accredited facility. Procedures were in accordance with NIH guidelines concerning care, use and euthanasia, including The Guide to the Care and Use of Laboratory Animals (National Research Council of the National Academies) and American Veterinary Medical Association Guidelines for the Euthanasia of Animals. Mice were anesthetized (IP) with ketamine (100mg/kg) and xylazine (10mg/kg). Mice were euthanized following pentobarbital overdose (200mg/kg IP) followed by bilateral thoracotomy or by carbon dioxide asphyxiation followed by cervical dislocation.

*Cd36*°*/Ldlr*° and *Ldlr*° mice were derived from a cross between *Cd36*° (created in our lab [40], 10x backcrossed to C57Bl/6J) and *Ldlr*° mice (10x backcrossed to C57Bl/6J, The Jackson Laboratory, B6.129S7-*Ldlr* ^*tm1Her*^/J, strain #002207). The double heterozygotes were crossed again to the *Ldlr*° background and *Cd36* heterozygote/*Ldlr*° mice were interbred to obtain the two background matched littermate derived strains used in the study. Mice were genotyped by polymerase chain reaction (PCR) on isolated tail DNA for *Cd36* as previously described, [31] and for *Ldlr* as recommended on the Jackson Laboratory website.

*Tlr2*° mice were from The Jackson Laboratory (B6.129-*Tlr2* ^*tm1Kir*^/J, strain #004650) and are C57Bl/6J congenic.

### *Porphyromonas gingivalis* Infection and Western Diet

Pg bacteria were grown under anaerobic conditions (Mitsubishi AnaeroPak anaerobe jar 2.5L, Thermo-Scientific R685025/AnaeroPack-Anaero System Thermo-Scientific R681001) in Schaedler broth containing vitamin K_1_ for 24–48 hours. To create the *in vivo* periodontal disease model, saturated cultures of Pg (~2 x 10^9^ CFU/ml) were resuspended in saline containing 2% carboxymethylcellulose (as a thickener to promote adherence) prior to oral inoculation of mice, using a modification of the method of Lalla, *et al*. [41]. Briefly, 200μl of bacterial suspension was administered using a micropipette at the gingival margin throughout the mouth and colorectal area every other day for 14 days. Mice are copacetic, and spreading bacteria in the rectal area promotes the cycle of infection. Mice were anesthetized (IP) with ketamine (100mg/kg) and xylazine (10mg/kg). Control mice received vehicle alone. The Western diet (Harlan Teklad 88317, 21% fat, 0.15% cholesterol, no cholate, irradiated) was begun simultaneously with the first inoculation and continued for 12 weeks.

For detection of bacteria in the lesion, the following primers were used: P1: 5’-AGG CAG CTT GCC ATA CTG CG-3’; P2: 5’-ACT GTT AGC AAC TAC CGA TGT-3’ [42]. The PCR protocol was 94°C x 30 seconds, 52°C x 1 minute, 72°C x 1 minute for 35 cycles.

### Periodontal Disease Assessment

**Fig 1:**
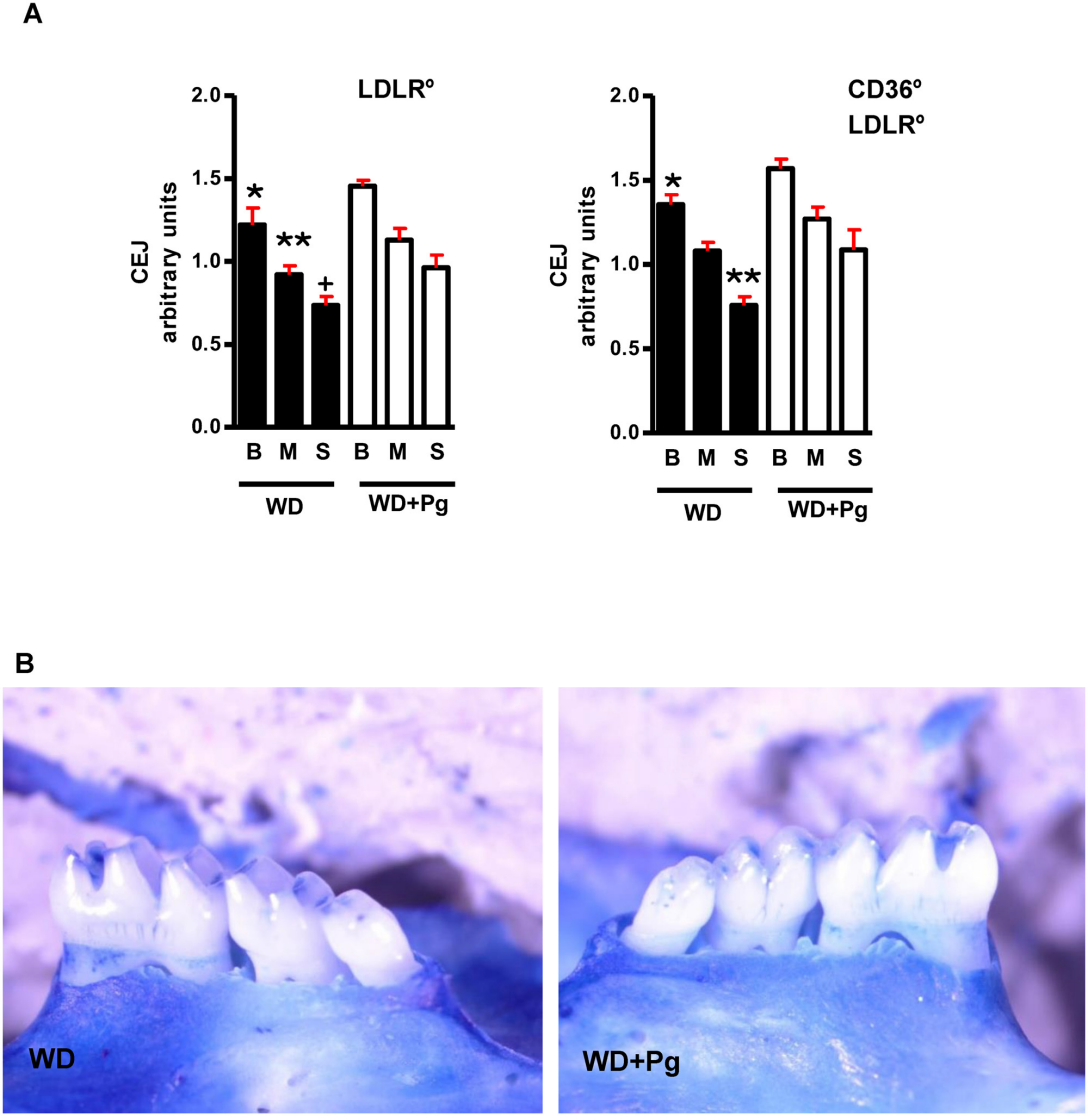
Pg infection increases the distance from the bone to the CEJ in a similar fashion in *Ldlr*° and *Cd36*°*/Ldlr*°mice. **A**. Methylene blue stained teeth were digitally imaged and the distance from the bone to the CEJ determined for each of the upper and lower big (B), middle (M) and small (S) molars (n = 11/group). *Ldlr*° (left panel) and *Cd36*°*/Ldlr*° (right panel) infected mice showed increased bone to CEJ distance compared with uninfected mice of the same strain. *,**,^+^ p<0.05 compared with corresponding tooth in Pg treated mice. **B**. Representative images from infected and uninfected mice.

Mandibles were isolated at sacrifice by pentobarbital overdose (200mg/kg, IP), defleshed after boiling, rinsed in 1% bleach, stained with 1% methylene blue and photographed under a Leica dissecting microscope (200x magnification) equipped with a digital camera. Where the cementum meets the enamel is termed the cemento-enamel-junction (CEJ), and the distance from the CEJ to the bone was measured as an indication of PD (average of 2–3 measurements/tooth for each upper and lower molar in a blinded fashion) [43,44]. This distance increases as there is bone resorption as a result of PD. Measurements were accomplished using Adobe Photoshop software and the “ruler” tool in arbitrary units for comparison. PG infected *Ldlr*° and *Cd36*°*/Ldlr*° mice had a similar increase in the distance from the CEJ to the bone compared with uninfected controls (Fig 1A). Representative images are shown (Fig 1B).

### Plasma Analyses

One week before sacrifice, mice were fasted overnight and tail vein blood collected in EDTA (5mM/L K_2_EDTA final concentration) for plasma analysis of triacylglycerol and non-esterified fatty acids. Mice were anesthetized (IP) with ketamine (100mg/kg) and xylazine (10mg/kg) and pain was relieved after the procedure by applying Kwikstop with benzocaine. Plasma was also collected at sacrifice (pentobarbital IP 200mg/kg) for total cholesterol, IL6 and IFNγ assays. Total cholesterol, triacylglycerol and non-esterified fatty acid concentrations were determined using kits from Wako or Pointe Scientific, Inc. as per the manufacturer’s instructions. IL6 and IFNγ were assayed by ELISA (R&D Systems).

For lipoprotein analysis, male mice (n = 5-8/group) were fasted overnight after 8 weeks Western diet feeding and tail vein blood collected in EDTA (5mM K_2_EDTA final concentration) as above. Plasma lipoprotein analyses were accomplished by fast protein liquid chromatography of 10 μL aliquots on two Superose 6 columns with continuous online detection of cholesterol in the eluent [45,46]. Results (mean ± S.E.) are expressed as percentage distribution of the specific lipoprotein relative to the total.

### Morphometry

Morphometry was performed as previously described [30–34]. Briefly, mice (n = 10-14/group) were sacrificed by pentobarbital overdose (200mg/kg, IP) and perfused with 10ml PBS and 5ml buffered formalin (FormaldeFresh). The entire aorta from the heart, extending 2-3mm after bifurcation of the iliacs and including the subclavian, right and left carotid arteries was dissected from the animal and post-fixed in FormaldeFresh. Aortae were stained with oil red O for 30 minutes and de-stained with methanol for 1–2 minutes with agitation. Individual aortae were placed open on a microscope slide, covered with a coverslip and hydrated with PBS. The aortae were then digitally scanned and percent lesion area determined using Adobe Photoshop software. Lesion area is expressed as percent of total aortic area ± S.E.

For assessment of morphological composition of lesions, 10μm aortic sinus lesion sections were cut at the level of the valve leaflets and stained with oil red O or trichrome stain, in collaboration with the laboratory of Jonathan D. Smith (Department of Cell Biology, Cleveland Clinic) [30]. Cellular and collagen content of digitally scanned sections were determined using Adobe Photoshop software.

### Macrophage Isolation

Macrophages were harvested by peritoneal lavage into PBS after sacrifice (CO_2_ asphyxiation) of gender and age matched mice. For elicitation, mice were injected IP with 2ml sterile 4% Brewer’s thioglycollate medium 4 days prior to sacrifice. Macrophages were cultured in DMEM containing 10% fetal bovine serum and 1% penicillin/streptomycin.

### Flow Cytometry and Bacteria Labeling

Flow cytometry analyses were performed on a Becton-Dickinson FACScan or LSR II (LRI Core Facility, Cleveland Clinic). Elicited peritoneal macrophages were incubated with Syto 17 labeled Pg bacteria and after extensive washing, interaction between bacteria and macrophages was analyzed by flow cytometry using Texas Red filters. Monoclonal anti-CD36/SRB2 antibody and isotype control were used in the blocking experiment at a concentration of 5 μg/ml. Levels of TLR2 were assessed by flow cytometry using anti-mouse TLR2 antibody (1:1000 dilution) or isotype control after incubation with FcR Blocking Reagent, and followed by Alexa Fluor 488 conjugated secondary antibody.

Bacteria were labeled with 50nM Syto 17 in saline for 30 minutes, (as per the manufacturer’s instructions) then washed extensively before incubation with 1x10^6^ elicited peritoneal macrophages for the indicated times. Bacteria were added at ~10:1 ratio to macrophages.

### IL1β Generation

Macrophages were plated at a density of ~0.5x10^6^/well of a 12 well plate. Non-adherent cells were washed away and cells were cultured for at least 24 hours prior to experiments. The morning of the experiment, the cells were fed with DMEM + 10% FBS. Inhibitors were added 30 minutes prior to PgLPS. Cells were treated with PgLPS overnight (16 hours) followed by 2 hour incubation with 5mM ATP. Unlike what has been reported for monocytes, ATP is essential for macrophage IL1β generation [47]. Supernatants were collected and frozen at -20°C for IL1β ELISA (BioLegend, Mouse IL1β ELISA Max Standard, 432602) and wells were treated with lysis buffer and scraped for determination of protein concentration. IL1β analysis was done in quadruplicate and repeated by separate experiment at least once.

The lysis buffer consisted of 20mM Tris-HCl, pH 7.4, 150mM NaCl, 1mM EDTA, 1mM EGTA. Inhibitors were used at the following concentrations: CAPE and Resveratrol, 100μM; DPI, 10μM; z-VAD-FMK and z-YVAD-FMK, 20μM; APDC and TMB8, 50μM; A740003, 200μMm; L-NAME, 1mM. For inhibitors that were only soluble in DMSO, stock solutions were prepared at 100x and a control well with the same final concentration of DMSO without inhibitor was included in the experiment.

OxLDL was added at a final concentration of 25 or 50μg/ml, as indicated in the figure legends. KOdiA-PC was added at a concentration of 10μg/ml.

### Foam Cell Assay

Macrophages were plated on sterile coverslips at a density of ~0.25x10^6^/well of a 12 well plate. Experiments were commenced after removal of non-adherent cells and culture for at least 24 hours. Cells were treated as indicated with PgLPS and/or oxLDL (25μg/ml) in DMEM + 1% FBS as indicated in the figure legends, then washed with PBS, fixed in 1% paraformaldehyde and stained with oil red O. After washing with PBS, coverslips were inverted on slides and cells were imaged using a Leica microscope equipped with a digital camera at 200x magnification. For quantification, 15 random fields were assessed. A relatively low concentration of oxLDL was used such that an increase in foam cells would be readily apparent.

### NFκB/SEAP Assay

Raw-Blue cells (Invivogen), which are a derivative of RAW 264.7 cells, a mouse macrophage-like line containing a stably incorporated NFκB reporter construct, were used to assess NFκB activation indirectly by measuring secreted alkaline phosphatase activity (SEAP) using the QuantiBlue reagent, as per the manufacturer’s (InvivoGen) instructions. We assessed activation by multiple concentrations of PgLPS (data not shown) and found that 0.1μg/ml was sufficient for robust, consistent activation (abs 635 = ~0.5 within 45 minutes), without rapid color saturation, and used this concentration throughout. Cells were pre-treated for 15 minutes with anti-TLR2 antibody (0.5μg/ml final) or anti-CD36 antibody (12.5μg/ml final). LDL and oxLDL were added 30 minutes prior at a concentration of 25μg/ml. These concentrations of anti-CD36 antibody, LDL and oxLDL were also used in experiments demonstrating decreased pyroptosis. Cells were pre-treated with thrombospondin-1 (TSP) for 15 minutes at a concentration of 50–500nm (a range that has been shown to inhibit angiogenesis). Although not shown, we also pre-treated with TSP for 30 minutes, 2 hours, or treated cells with TSP and PgLPS simultaneously. No differences were observed in SEAP activity.

### Statistical Analysis

Results are presented as mean ± S.E. Results were subjected to the D’Agostino and Pearson normality test. Those that passed normality were then assessed by two-tailed t-test or one-way ANOVA followed by Bonferroni’s Multiple Comparison Test. If normality was not met, Mann-Whitney analysis was performed (for comparison between 2 groups) or a Kruskal-Wallis analysis followed by Dunn’s Multiple Comparison Test was performed. Statistical significance was set at p≤0.05.

## Results

### Atherosclerosis lesion burden is increased in Pg infected *Ldlr*° mice and the increase is mediated by CD36/SR-B2

**Fig 2:**
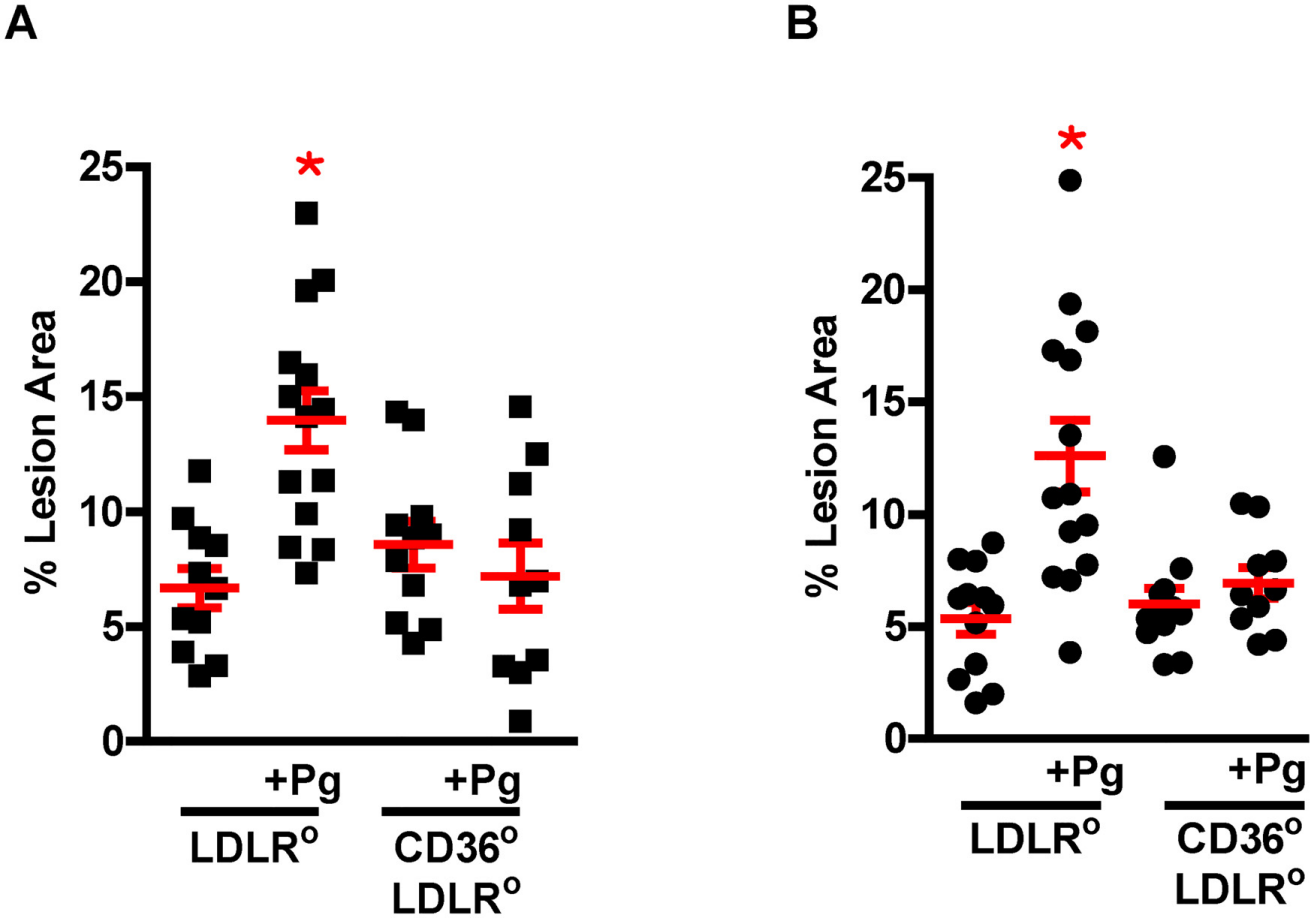
CD36/SR-B2 is essential to increased lesion burden. Lesion area in the aortic tree of Pg infected or uninfected male (left) and female (right) *Ldlr*° and *Cd36*°*/Ldlr*° mice. Increased atherosclerosis as a result of Pg infection was CD36/SR-B2 dependent. One way ANOVA, p< 0.0001. Bonferroni’s Multiple Comparison Test, *p<0.05 vs all other groups (n = 10–14 mice/group).

**Fig 3:**
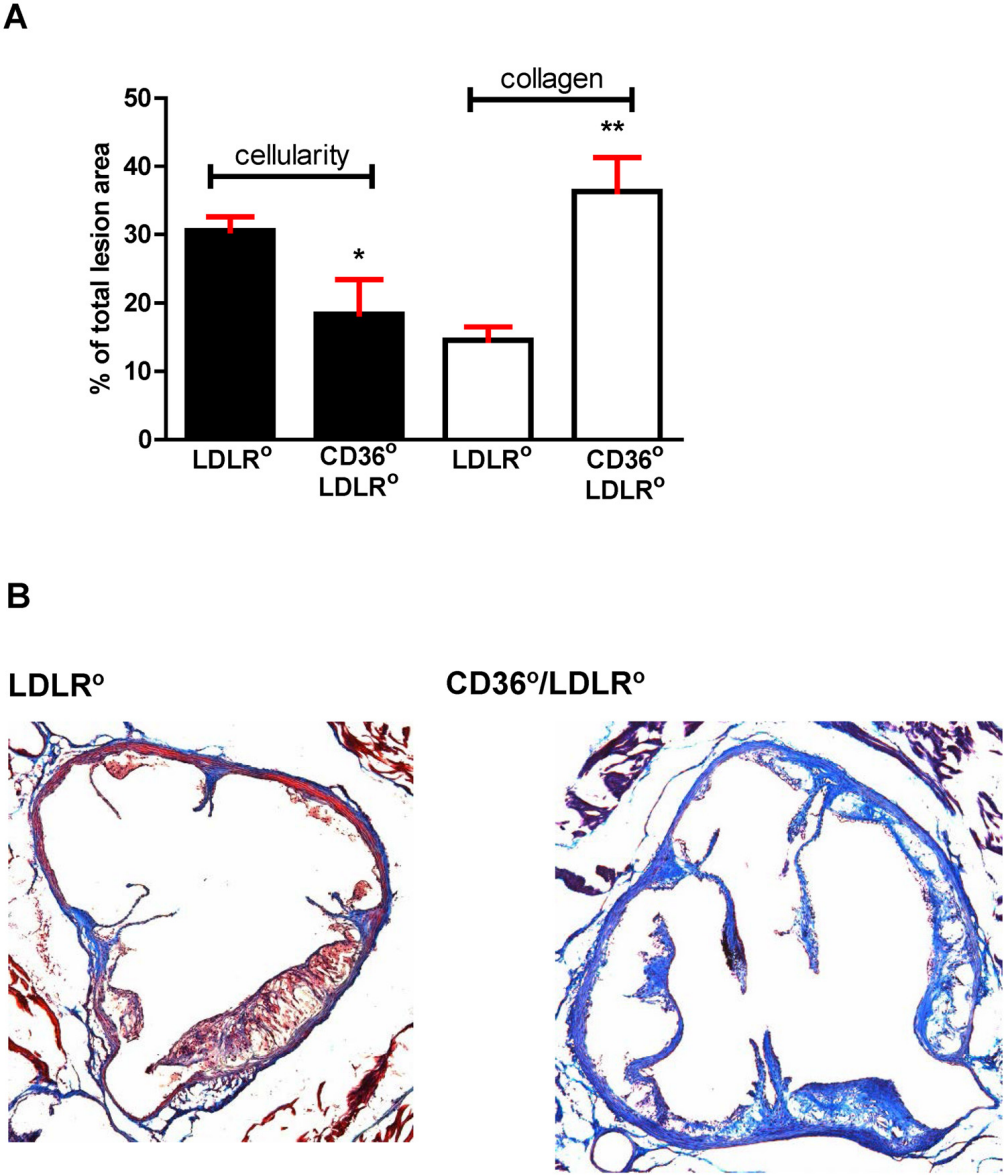
Morphological assessment of aortic sinus lesions in Pg infected mice. **A**. Lesions from Pg infected *Ldlr*° mice had greater cellularity and reduced collagen content compared with those from *Cd36*°*/Ldlr*° mice (n = 13 lesions/group from 5–6 mice). Student’s two-tailed t-test, *p<0.05, **p<0.0005. **B**. Representative lesions stained with trichrome. Red = cells; Blue = collagen.

There were no differences in lesion area between male WD fed *Ldlr*° and *Cd36*°*/Ldlr*° mice (Fig 2A, 7.97% ± 1.06 *vs* 7.63% ± 1.3), or female WD fed *Ldlr*° and *Cd36*°*/Ldlr*° mice (Fig 2B, 5.6% ± 0.8 *vs* 6.72% ± 1.37) which confirms our previous study showing that atherosclerosis in this model is CD36/SR-B2 independent [33]. Both male (13.96% ± 1.28) and female (12.6% ± 1.58) *Ldlr*° mice infected with Pg showed significantly increased lesion burden. In contrast, there was no increase in lesion burden in male (7.2% ± 1.45) or female (6.94% ± 0.7) *Cd36*°*/Ldlr*° mice infected with Pg compared with uninfected mice. Thus, the increased lesion in Pg infected *Ldlr*° mice was entirely dependent upon CD36/SR-B2. This confirms our previous work that inflammation in this model leads to a CD36/SR-B2-dependent component of lesion development [33]. As in our previous study, we found that lesions from *LDLR*° mice contained more macrophages than those from *Cd36*°*/Ldlr*° mice [33] (data not shown). Lesions from *LDLR*° mice infected with Pg were more cellular and had less collagen than infected *Cd36*°*/Ldlr*° mice (Fig 3). These data show an essential role for CD36/SR-B2 in transmitting Pg effects to the vasculature *in vivo*.

In order to determine if there was direct colonization of the lesion (which has been controversial [48,49]), at sacrifice we isolated lesion at the juncture of the heart and aorta for PCR analysis using primers specific to Pg. No band corresponding to Pg amplified. Control primers showed the DNA templates were intact (data not shown). These data suggest that live bacteria do not persist in the lesion in this model (but do not rule out the presence of PgLPS or early infection that does not persist).

### Physiological risk factors do not account for differences in lesion burden

**Fig 4:**
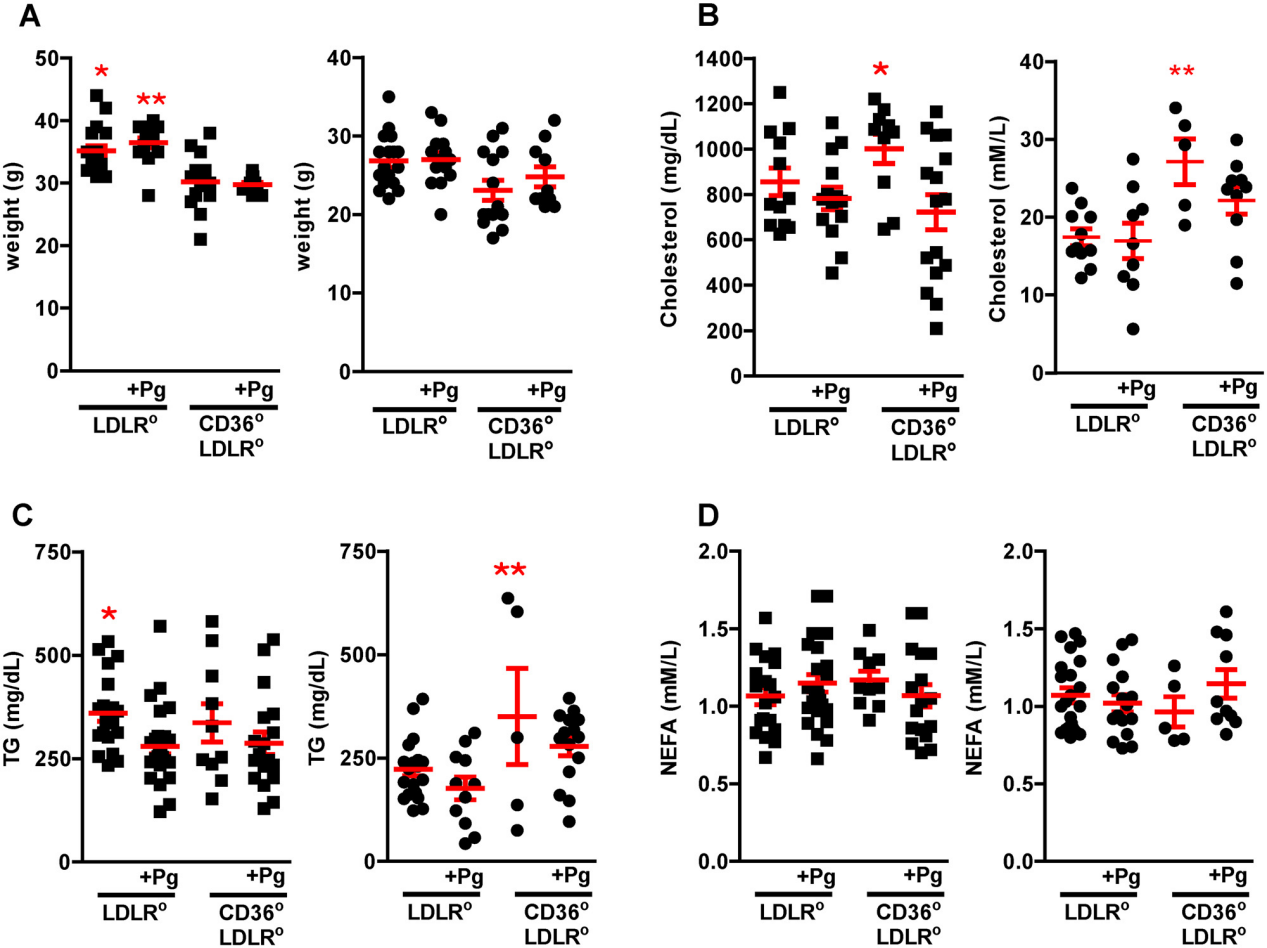
Weight and plasma analyses do not explain differences in lesion burden. **A-D**. Weights and plasma analyses from Pg infected or uninfected male (left, squares) and female (right, circles) *Ldlr*° and *Cd36*°*/Ldlr*° mice (n = 5–26 mice/group). **A**. Uninfected and Pg infected male *Ldlr*° mice were significantly heavier than uninfected and Pg infected *Cd36*°*/Ldlr*° male mice. Kruskal-Wallis Test, p<0.0001, Dunn’s Multiple Comparison Test, *, **p<0.05. **B**. Uninfected *Cd36*°*/Ldlr*° male mice had significantly higher plasma total cholesterol levels compared with infected *Cd36*°*/Ldlr*° males. One way ANOVA, p< 0.05. Bonferroni’s Multiple Comparison Test, *p<0.05. Uninfected *Cd36*°*/Ldlr*° females had higher total plasma cholesterol levels compared with uninfected and Pg infected female *Ldlr*° mice. One way ANOVA, p≤ 0.01. Bonferroni’s Multiple Comparison Test, **p<0.05. **C**. Uninfected male *Ldlr*° mice had significantly higher plasma triacylglycerol levels compared with infected *Ldlr*° male mice. Kruskal-Wallis Test, p<0.05, Dunn’s Multiple Comparison Test, *p<0.05. Uninfected female *Cd36*°*/Ldlr*° mice had significantly higher plasma triacylglycerol levels compared with Pg infected *Ldlr*° female mice. One way ANOVA, p< 0.0005. Bonferroni’s Multiple Comparison Test, **p<0.05. **D**. Levels of plasma non esterified fatty acids did not differ in uninfected and Pg infected male or female *Ldlr*° and *Cd36*°*/Ldlr*° mice.

**Fig 5:**
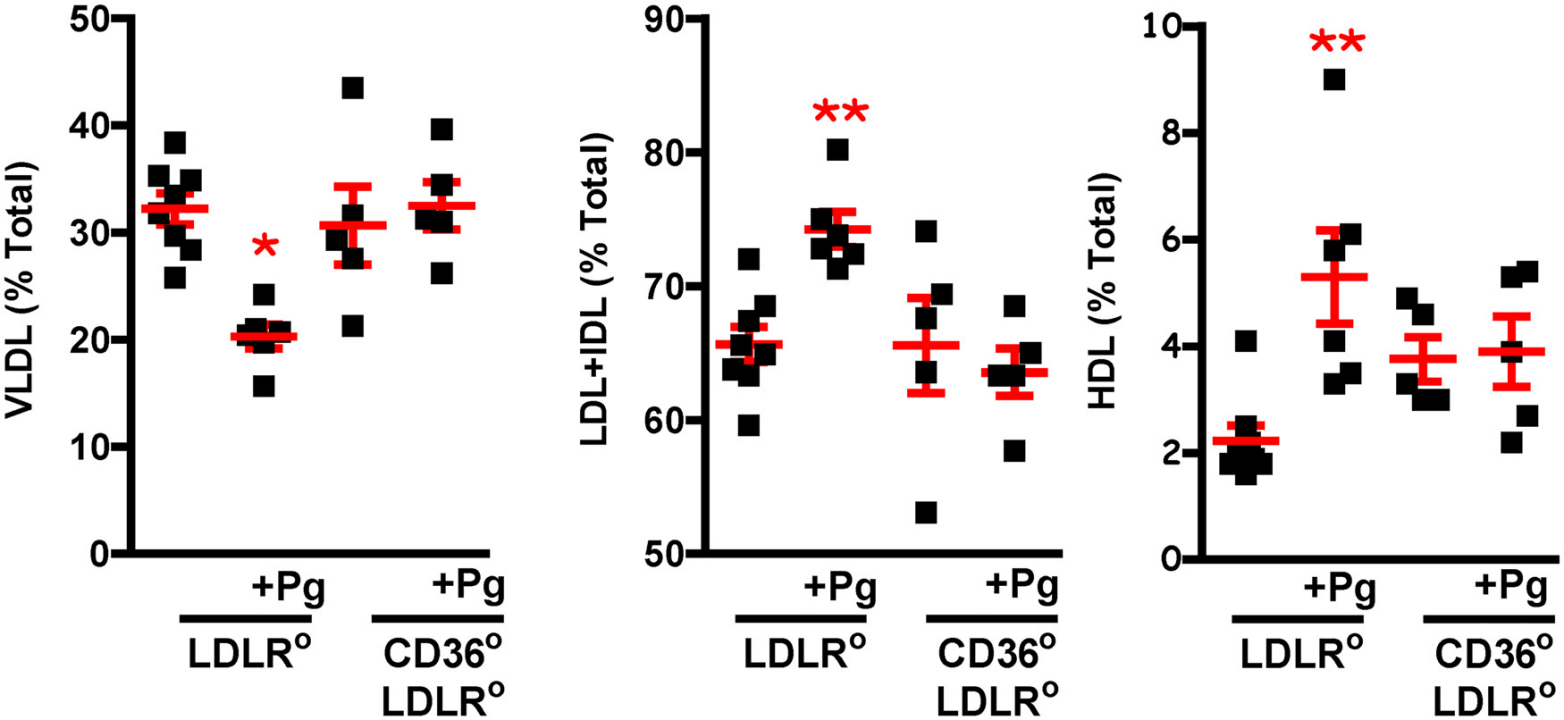
Lipoprotein analysis does not explain differences in lesion burden. Lipoprotein analysis of plasma from uninfected and Pg infected male *Ldlr*° and *Cd36*°*/Ldlr*° mice after 6 weeks of Western diet feeding. Pg infected *Ldlr*° mice had significantly lower levels of plasma VLDL and significantly higher levels of plasma LDL/IDL compared with uninfected and infected *Cd36*°*/Ldlr*° mice (n = 5–8 mice/group). One way ANOVA, *p< 0.005, **p<0.01. Bonferroni’s Multiple Comparison Test, *,**p<0.05. Pg infected *Ldlr*° mice had significantly higher levels of plasma HDL compared with uninfected *Ldlr*° mice. One way ANOVA, *p< 0.01. Bonferroni’s Multiple Comparison Test, ^+^p<0.05.

Although uninfected *Cd36*°*/Ldlr*° female mice had higher plasma cholesterol and triacylglycerol levels compared with all other females, this did not lead to an increase in lesion burden (Fig 4). *Ldlr*° males were heavier than *Cd36*°*/Ldlr*° males, but infected and uninfected *Ldlr*° males had marked disparity in lesion burden despite similar weight, suggesting that weight was not a factor in lesion development (Fig 4A). Uninfected *Cd36*°*/Ldlr*° males had higher levels of plasma cholesterol (Fig 4B), while uninfected *Ldlr*° males had higher triacylglycerol levels (Fig 4C). Non-esterified fatty acid levels were similar in all groups (Fig 4D). Lipoprotein profiles showed that Pg infected *Ldlr*° male mice had more IDL/LDL but this was offset by decreased VLDL and increased HDL (>2x) (Fig 5). Maekawa *et al*. [50] associated differences in lipoprotein profile to atherosclerosis lesion burden in a Pg mediated PD model in *Apoe*° mice. Our data differ, and this may be a reflection of the substantial lipoprotein disparities between the *Apoe*° and the *Ldlr*° models. A more recent study using a chronic oral Pg infection model in the *Apoe*° also did not find a correlation with lipoprotein profile [51]. Overall, although there were significant changes in some physiological risk factors in our study, they did not explain differences in lesion burden.

**Fig 6:**
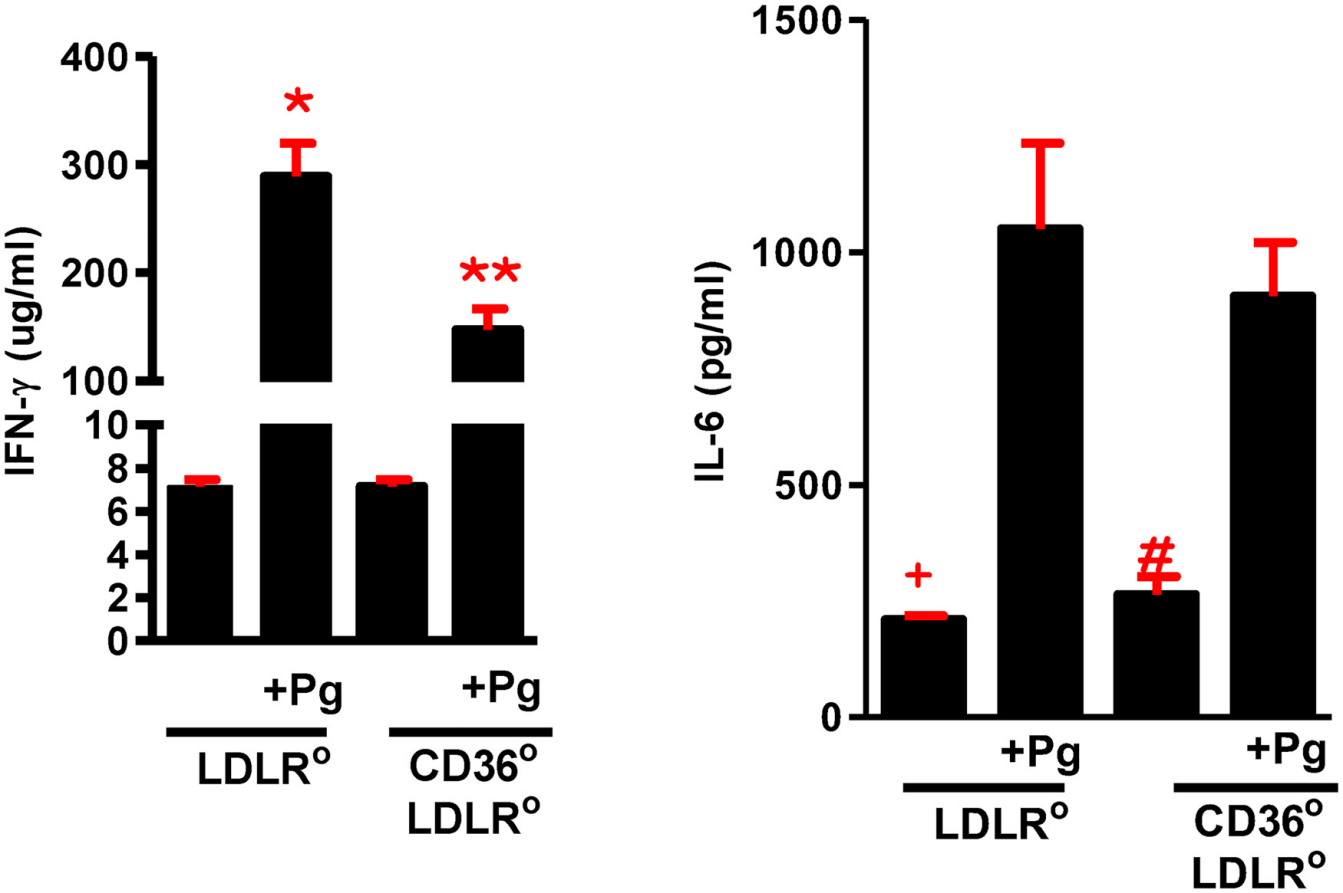
Plasma levels of IFNγ and IL6. Pg infected *Ldlr*° and *Cd36*°*/Ldlr*° mice had higher levels of IFNγ and IL6 compared with uninfected mice. One way ANOVA, p≤ 0.0001. Bonferroni’s Multiple Comparison Test, *,**, ^+^, ^#^p<0.05 compared with uninfected counterpart. Infected *Cd36*°*/Ldlr*° mice had significantly lower levels of IFNγ compared with infected *Ldlr*° mice, n≥3 mice/group. One way ANOVA, p≤ 0.0001. Bonferroni’s Multiple Comparison Test, ^&^ p<0.05 compared with Pg infected *Ldlr*° mice.

We measured serum levels of IFNγ and IL6, two cytokines associated with Pg infection and PD [52–54]. Both cytokines were significantly elevated in infected compared to uninfected mice; IFNγ was also higher in *Ldlr*° compared to *Cd36*°*/Ldlr*° mice (Fig 6). Increase in these cytokines further validates the PD model.

### Pg associates with macrophages from WT and *Cd36*° mice similarly

**Fig 7:**
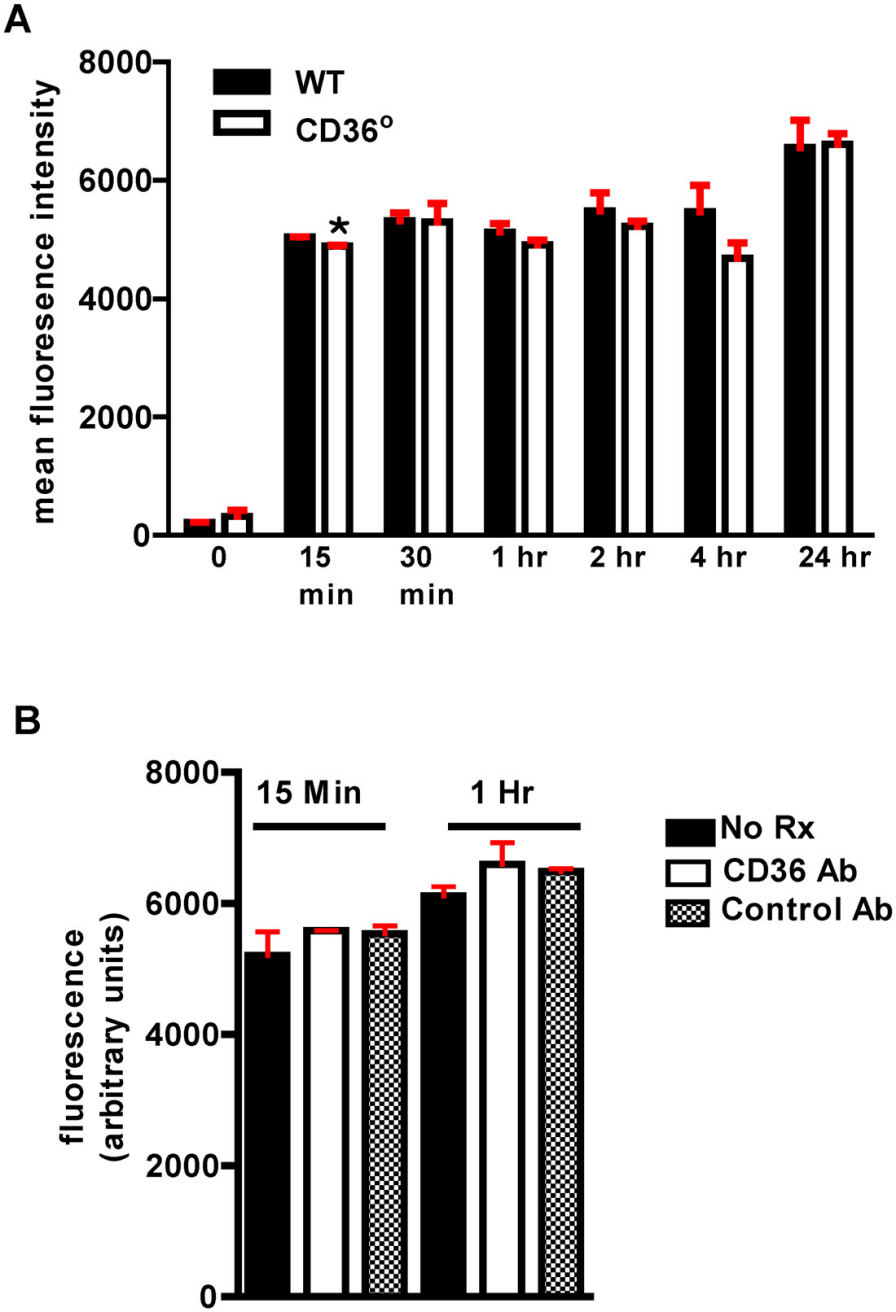
Pg bacteria interaction with macrophages is unaffected by CD36. **A**. Interaction of Syto 17 labeled Pg bacteria with macrophages from WT and *Cd36*° mice showed no significant differences by flow cytometry analysis after 15 minutes co-incubation. *p<0.005, one-tailed unpaired t-test. **B**. Antibody to CD36 did not affect interaction of Syto 17 labeled Pg bacteria with WT macrophages. Experiment was performed in triplicate and repeated once.

To rule out differences in Pg infection of macrophages as a potential mechanism for differences in atherosclerosis, we assessed *in vitro* macrophage-bacteria interactions. Flow cytometric analysis of Syto 17 labeled Pg bacteria incubated with WT and *Cd36*° macrophages for up to 24 hours showed a small but significant difference in mean fluorescence intensity at 15 minutes, but at no other time point (Fig 7A). WT macrophages incubated with labeled bacteria in the presence of antibody specific for murine CD36/SR-B2 or isotype control for 15 minutes and 1 hour showed no significant differences in associated bacteria (Fig 7B). These data suggest that CD36/SR-B2 is not essential for macrophage uptake of Pg.

### Mechanistic studies show differential macrophage responses

**Fig 8:**
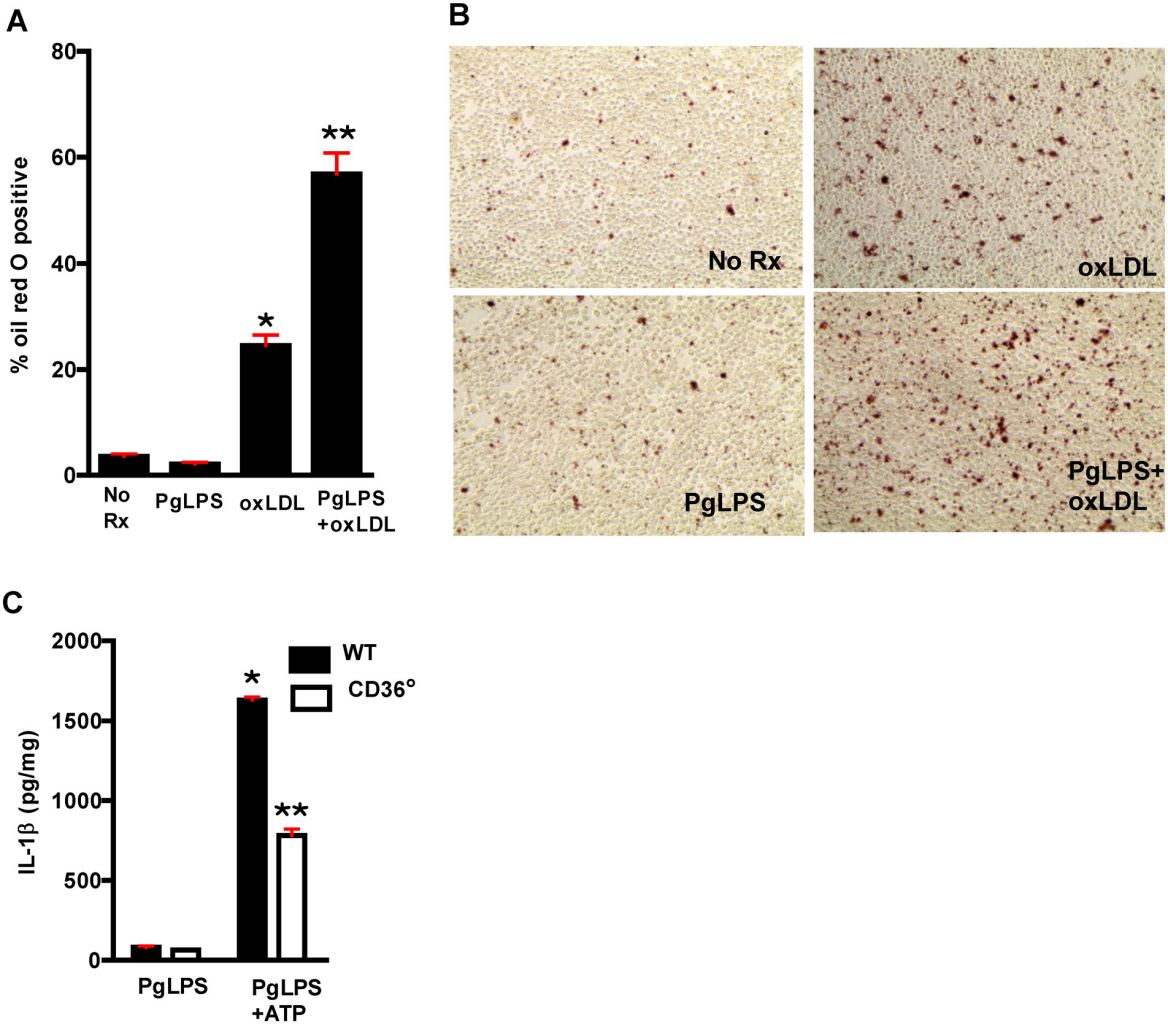
CD36/SR-B2 is essential to increased foam cell formation and IL1β generation in response to Pg. **A**. PgLPS significantly increases oxLDL-mediated accumulation of lipid and foam cell formation in primary macrophages. Cells were treated as indicated with PgLPS and/or oxLDL (25μg/ml) in DMEM + 1% FBS for 5 hours. One way ANOVA, p< 0.0001. Bonferroni’s Multiple Comparison Test, p<0.05. * significantly higher than no Rx, PgLPS. ** significantly higher than no Rx, PgLPS, oxLDL. Experiment was performed in triplet and repeated once. **B**. Representative images demonstrating increased foam cell formation in primary macrophages in the presence of PgLPS. **C**. Macrophages from *Cd36*° mice have a reduced IL1β response to PgLPS. Kruskal-Wallis Test, p<0.0001. Dunn’s Multiple Comparison Test p<0.05. Experiment was performed in quadruplicate and repeated three times.

WT and *Cd36*° macrophages were exposed *in vitro* to PgLPS, oxLDL or both, and then assessed for lipid uptake by oil red O staining. PgLPS significantly enhanced oxLDL uptake by more than 150% in WT macrophages and to a lesser extent in *Cd36*° macrophages (Fig 8A and 8B).

**Fig 9:**
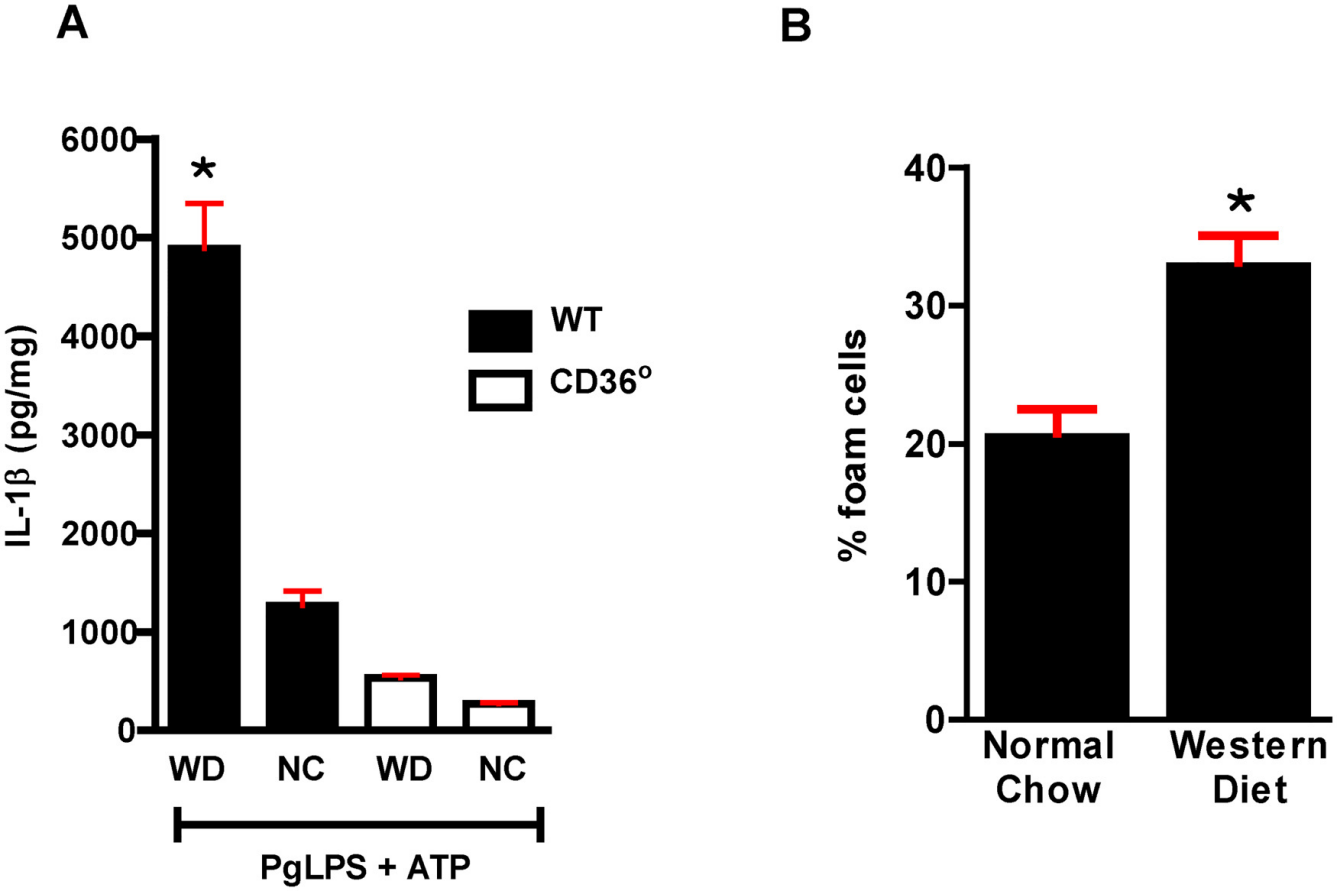
Western diet feeding influences responses to Pg. **A.** Macrophages from WT Western diet fed (WD) mice generate higher levels of IL1β in response to PgLPS compared with macrophages from normal chow (NC) fed WT mice and macrophages from WD or NC fed *Cd36*° mice. One way ANOVA, p< 0.0001. Bonferroni’s Multiple Comparison Test, *p<0.05 vs all other groups. Experiment was performed in triplet and repeated once. **B**. PgLPS+oxLDL lead to greater lipid accumulation and foam cell formation in macrophages from Western diet fed *Ldlr*° mice compared with macrophages from normal chow fed mice. Experiment was performed in triplet and repeated once. Student’s t-test, *p<0.0001.

PgLPS is a TLR ligand and CD36/SR-B2 has been shown to interact with TLRs, thus we tested the hypothesis that CD36/SR-B2 was involved in IL1β activation in response to PgLPS as a potential downstream effect of TLR signaling [55]. As shown in Fig 8C, absence of CD36/SR-B2 resulted in a significant decrease in IL1β generation by macrophages. We next investigated the effect of a high fat diet on responses to PgLPS. Isolated macrophages from WT mice fed the WD for 6 weeks showed significantly increased generation of IL1β compared with macrophages from normal chow (NC) fed mice and *Cd36*° mice fed either diet (Fig 9A). When incubated with oxLDL in the presence or absence of PgLPS, macrophages from WD fed *Ldlr*° mice showed increased foam cell formation (Fig 9B).

**Fig 10:**
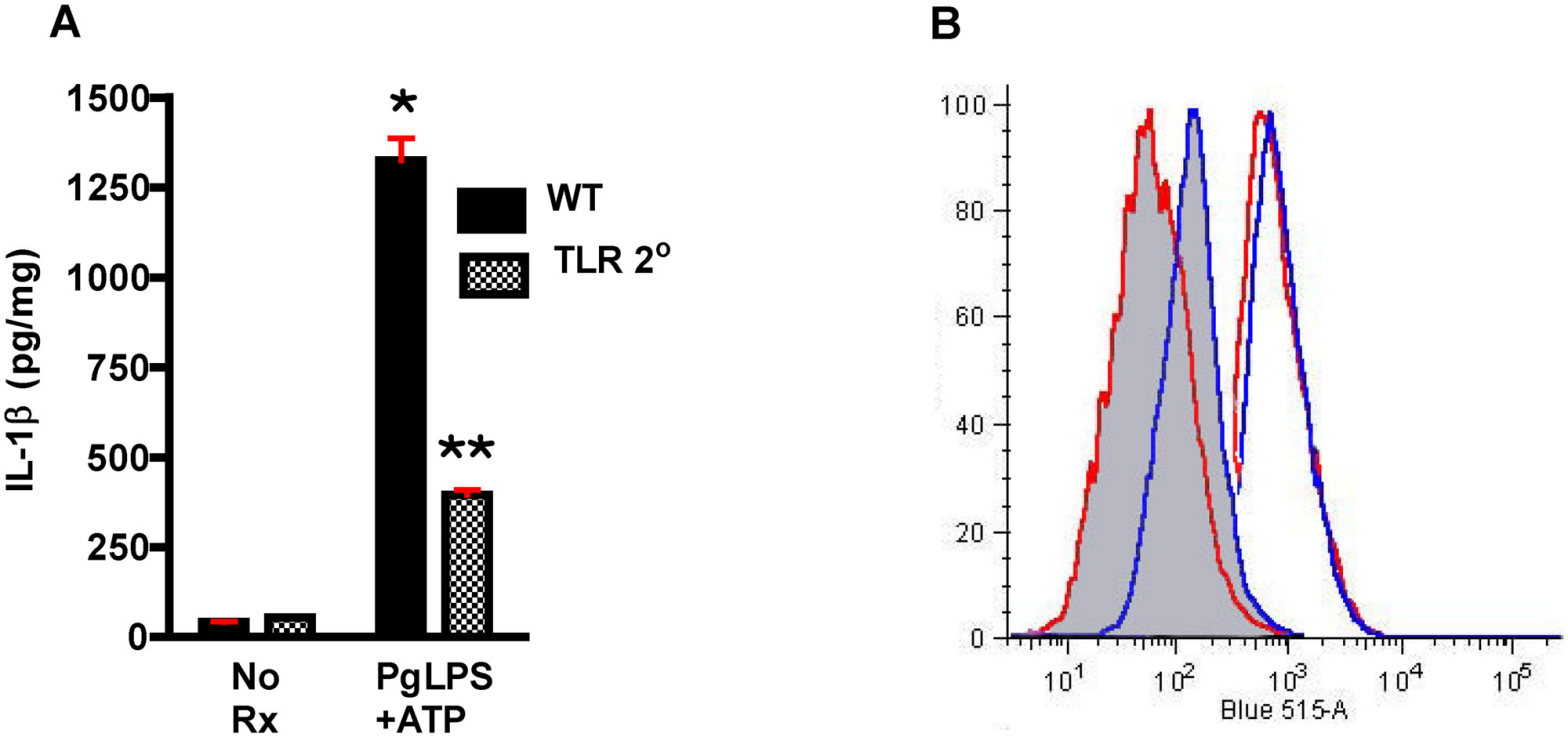
TLR2 plays a non-redundant role in IL1β generation in response to PgLPS. **A.** PgLPS mediated IL1β generation is reduced in macrophages from *Tlr2*° mice compared with macrophages from WT mice. One way ANOVA, p< 0.0001. Bonferroni’s Multiple Comparison Test, *,**p<0.05. Experiment was performed in quadruplicate and repeated twice. **B**. Membrane expression levels of TLR2 are similar on macrophages from WT (red) and *Cd36*° (blue) mice by flow cytometry analysis. Isotype control antibody is shown in the shaded plots, anti-CD36/SR-B2 antibody by the unshaded plots. Experiment was performed in triplicate and repeated twice.

PgLPS has been reported to be a ligand for both TLR2 and TLR4, although there is controversy as to whether it is the LPS itself or a contaminating bacterial lipoprotein [56,57]. Unlike TLR4, TLR2 has been shown to be pro-atherogenic and therefore we tested its involvement in the responses under study, using macrophages obtained from *Tlr2*° mice. We found marked reduction in generation of IL1β suggesting a non-redundant role for TLR2 in our system (Fig 10A). To be certain that modulation of TLR2 expression did not underlie the decrease in IL1β generation observed in *Cd36*° macrophages, we analyzed WT and *Cd36*° macrophages by flow cytometry and found similar expression levels of TLR2 (Fig 10B).

### PgLPS activates the NALP3 inflammasome

**Fig 11:**
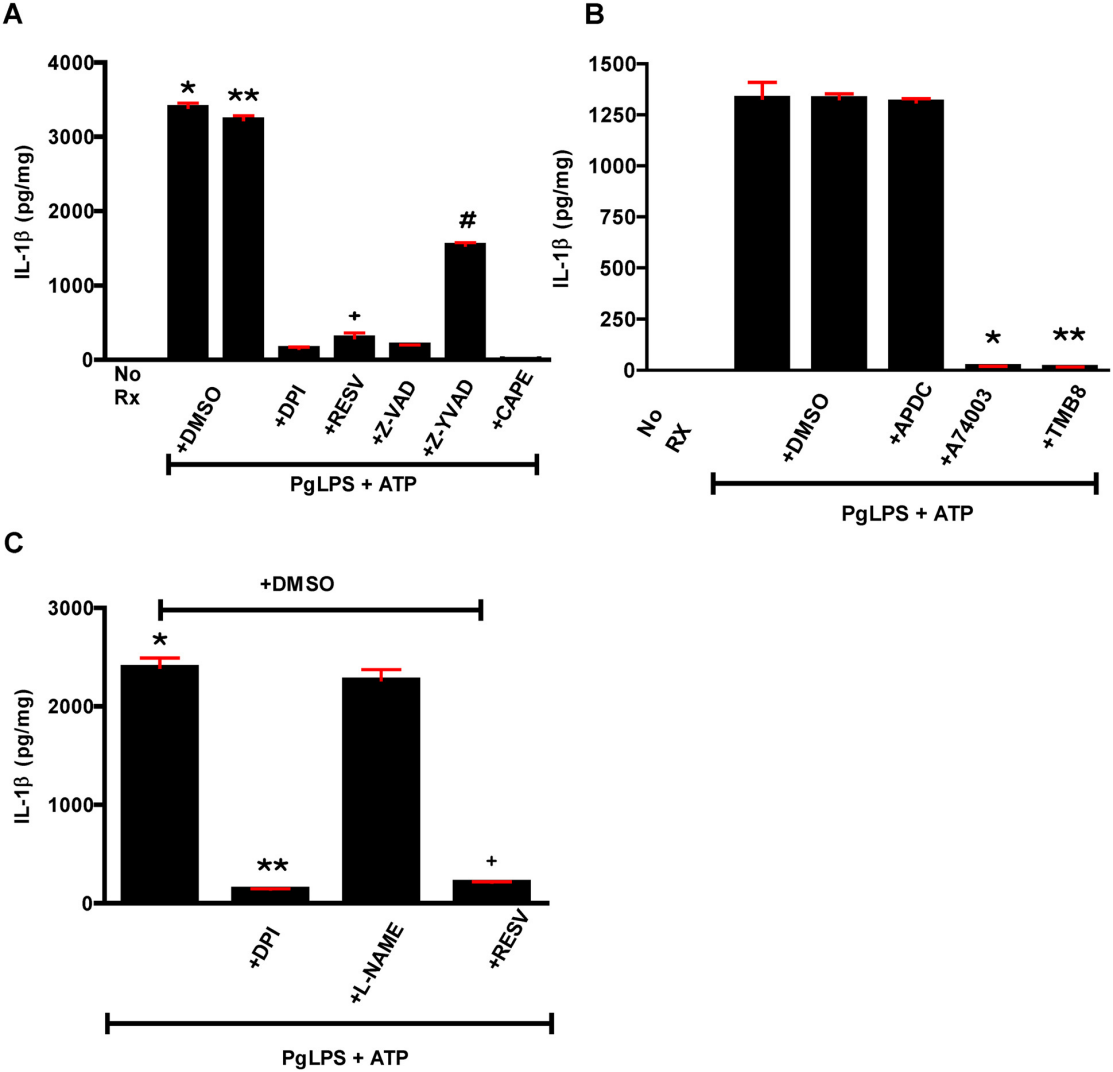
Inhibitor studies show that PgLPS mediated generation of IL1β is dependent upon inflammasome activation. **A-C.** NFκB nuclear translocation was blocked by CAPE; caspase1 activation was inhibited by the pan-caspase inhibitor Z-VAD-FMK and the specific inhibitor, Z-YVAD-FMK. Inhibitors and scavengers of ROS included DPI, resveratrol (RESV), APDC and L-NAME. Only those that inhibited NADPH oxidase affected IL1β generation. Blocking internal calcium stores release (TMB8) or the P2X7 potassium efflux channel (A740003) also inhibited IL1β generation. These data are consistent with inflammasome activation. **A.** One way ANOVA, p< 0.0001. Bonferroni’s Multiple Comparison Test, p<0.05. *, **PgLPS+ATP or PgLPS+ATP+DMSO *vs* no Rx, +DPI, +RESV, +Z-VAD, +Z-YVAD, +CAPE. ^+^PgLPS+ATP+RESV *vs* no Rx, +CAPE. ^#^ PgLPS+ATP+Z-YVAD *vs* DPI, +RESV, +Z-VAD, +CAPE. **B.** One way ANOVA, p< 0.0001. Bonferroni’s Multiple Comparison Test, p<0.05. *,**PgLPS+ATP+A740003 or PgLPS+ATP+TMB8 *vs* all groups except no Rx. **C.** One way ANOVA, p< 0.0001. Bonferroni’s Multiple Comparison Test, p<0.05. *PgLPS+ATP+DMSO *vs* all other groups except +L-NAME. ** PgLPS+ATP+DMSO+DPI *vs* all other groups except PgLPS+ATP+DMSO+RESV. ^+^PgLPS+ATP+DMSO+RESV *vs* all other groups except PgLPS+ATP+DMSO+DPI. Experiments were done in quadruplicate and repeated twice.

The classical mechanism for IL1β generation involves TLR mediated signaling through a myeloid differentiation primary response 88 (MyD88) pathway, NFκB activation, and transcription and translation of pro-IL1β protein [55,58]. Active IL1β is then generated through cleavage by caspase1. Caspase1 is itself produced as a pro-protein, and its activation in macrophages depends on assembly of the NACHT, LRR and PYD domains-containing protein 3 (NALP3) inflammasome [58,59]. In addition to this classical pathway, non-inflammasome mediated generation of IL1β has been described [60–65]. We investigated the involvement of NFκB and the NALP3 inflammasome in Pg-mediated IL1β generation using a series of inhibitors (Fig 11). None of the inhibitors affected cell viability at the concentrations used, and the data strongly support the canonical pathway for IL1β generation in the TLR2-CD36/SR-B2 response to PgLPS.

### CD36/SR-B2 ligands inhibit PgLPS mediated inflammasome activation

**Fig 12:**
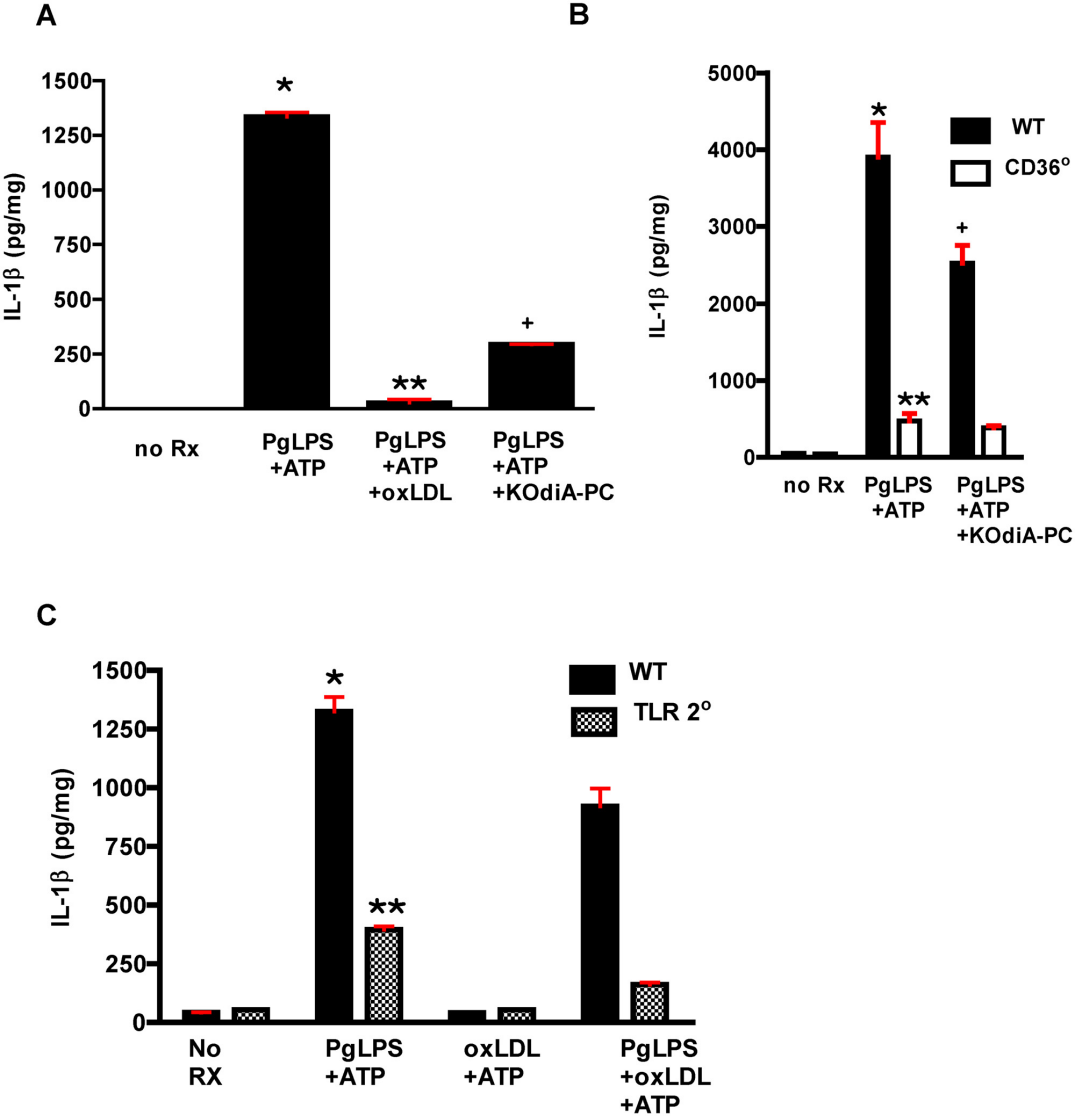
IL1β generation in response to Pg is inhibited by oxLDL in a CD36/SR-B2 dependent and TLR2 independent manner. **A.** Both oxLDL and the more specific CD36/SR-B2 ligand present within oxLDL, KOdiA-PC, inhibit IL1β generation in response to PgLPS in macrophages from WT mice. One way ANOVA, p< 0.0001. Bonferroni’s Multiple Comparison Test, p<0.05. *, + vs all groups; ** vs all groups except no Rx. Experiment was performed in quadruplicate and repeated twice. **B**. IL1β inhibition by KOdiA-PC is dependent upon CD36/SR-B2. No further inhibition is observed in macrophages from *Cd36*° mice. One way ANOVA, p< 0.0001. Bonferroni’s Multiple Comparison Test, p<0.05. *, + vs all groups; ** vs all groups except *Cd36*° treated with KOdiA-PC. Experiment was performed in quadruplicate and repeated twice. **C**. IL1β inhibition by oxLDL is independent of TLR2 expression. IL1β generation in response to PgLPS+ATP is inhibited in macrophages from *Tlr2*° mice. One way ANOVA, p< 0.0001. Bonferroni’s Multiple Comparison Test, p<0.05. *, ** vs all groups. Experiment was performed in quadruplicate and repeated twice.

Augmentation of IL1β generation and foam cell formation in cells from WD fed mice suggested that CD36/SR-B2 specific ligands on LDL created as a result of reactive oxygen and nitrogen species from inflammatory processes were involved [66,67]. To test this hypothesis directly, we incubated macrophages with oxLDL, the more specific CD36/SR-B2 ligand present in oxLDL, KOdiA-PC, and PgLPS *in vitro*. Both oxLDL and KOdiA-PC strongly inhibited Pg-mediated IL1β generation (Fig 12A). KOdiA-PC had no effect on residual IL1β generation in *CD36*° macrophages, suggesting that the effect was mediated by CD36/SR-B2 (Fig 12B). TLR2 was not necessary for the effect (Fig 12C). Interestingly, oxLDL could inhibit generation of IL1β after priming with PgLPS (Fig 12A and 12C), implying that oxLDL neither sequestered PgLPS nor hindered interaction of CD36/SR-B2 with PgLPS or TLR2.

### CD36/SR-B2 and TLR2 are necessary for PgLPS mediated NFκB activation; inhibition by oxLDL

**Fig 13:**
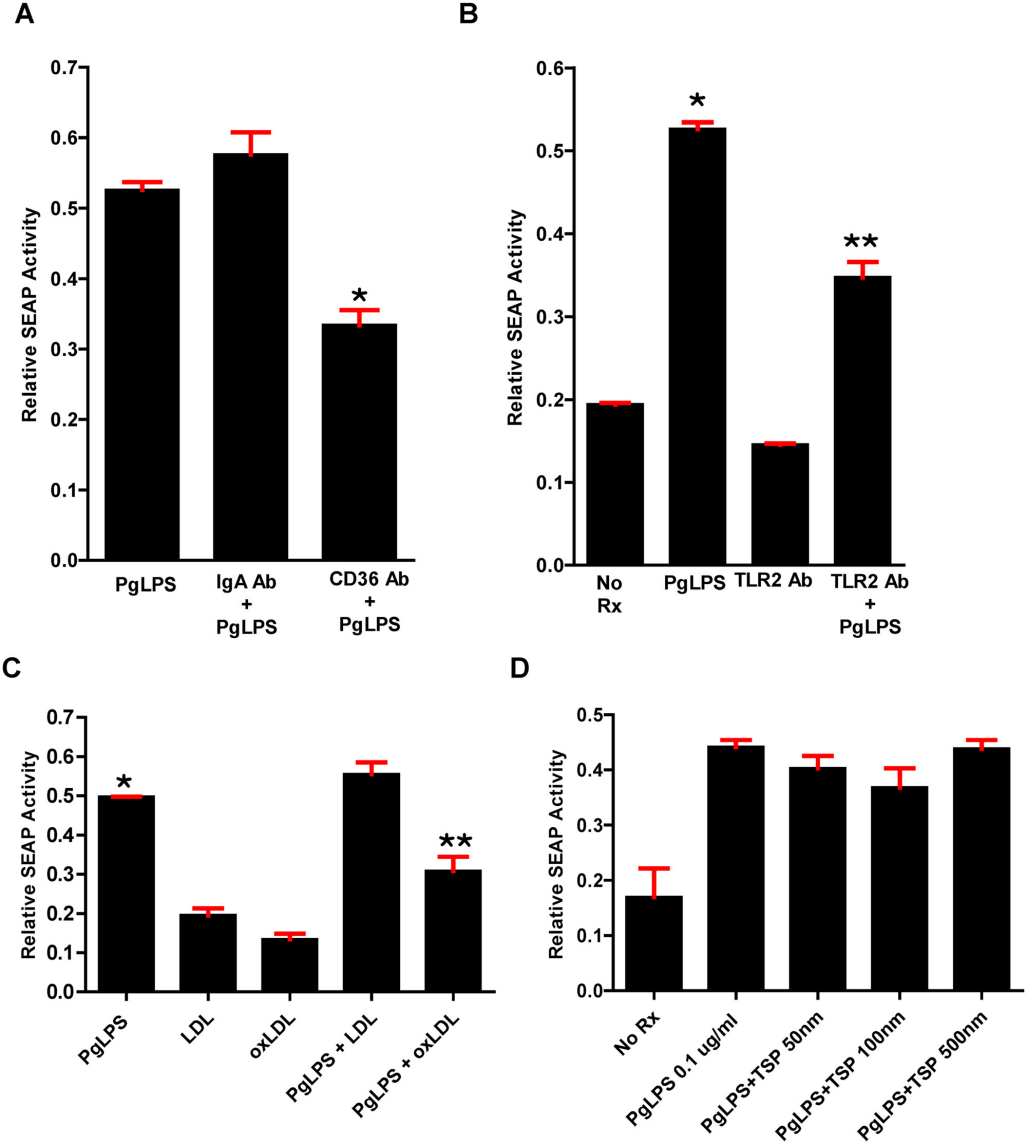
PgLPS induced NFκB activation is inhibited by antibody to CD36/SR-B2 and TLR2, and oxLDL. NFκB activation was assessed in RAW-Blue cells containing a secreted alkaline phosphatase (SEAP) reporter construct. **A**. Monoclonal antibody to CD36/SR-B2, but not control IgA significantly inhibited NFκB activation in response to PgLPS. One way ANOVA, p< 0.0001. Bonferroni’s Multiple Comparison Test, p<0.05. *vs all groups. Experiment was performed in triplicate and repeated three times. **B**. Polyclonal antibody to TLR2 significantly inhibited NFκB activation in response to PgLPS. Antibody alone could not activate NFκB at the concentration added. One way ANOVA, p< 0.005. Bonferroni’s Multiple Comparison Test, p<0.05. *,** vs all groups. Experiment was performed in triplicate and repeated twice. **C**. OxLDL but not LDL inhibits NFκB activation in response to PgLPS. One way ANOVA, p< 0.005. Bonferroni’s Multiple Comparison Test, p<0.05. * vs all groups except PgLPS+LDL; ** vs all other groups except LDL. Experiment was performed in triplicate and repeated twice. **D**. Pre-treatment with thrombospondin-1 (TSP) had no effect on NFκB activation in response to PgLPS. One way ANOVA, p> 0.05. Experiment was performed in triplicate and repeated twice.

Using SEAP activity as a marker of NFκB activation in RAW-Blue cells, a murine macrophage cell line, we found as expected that antibody to TLR2 inhibited activation of NFκB (Fig 13B). Interestingly, antibody to CD36/SR-B2 also inhibited NFκB activation. Thus CD36/SR-B2 plays an essential role in macrophage priming by PgLPS (Fig 13A). OxLDL, but not native LDL, or thrombospondin-1 (TSP), a ligand of CD36 that inhibits angiogenesis, inhibited PgLPS-mediated NFκB activation (Fig 13C and 13D).

### IL1β potentiates IL1β generation and macrophage foam cell formation

**Fig 14:**
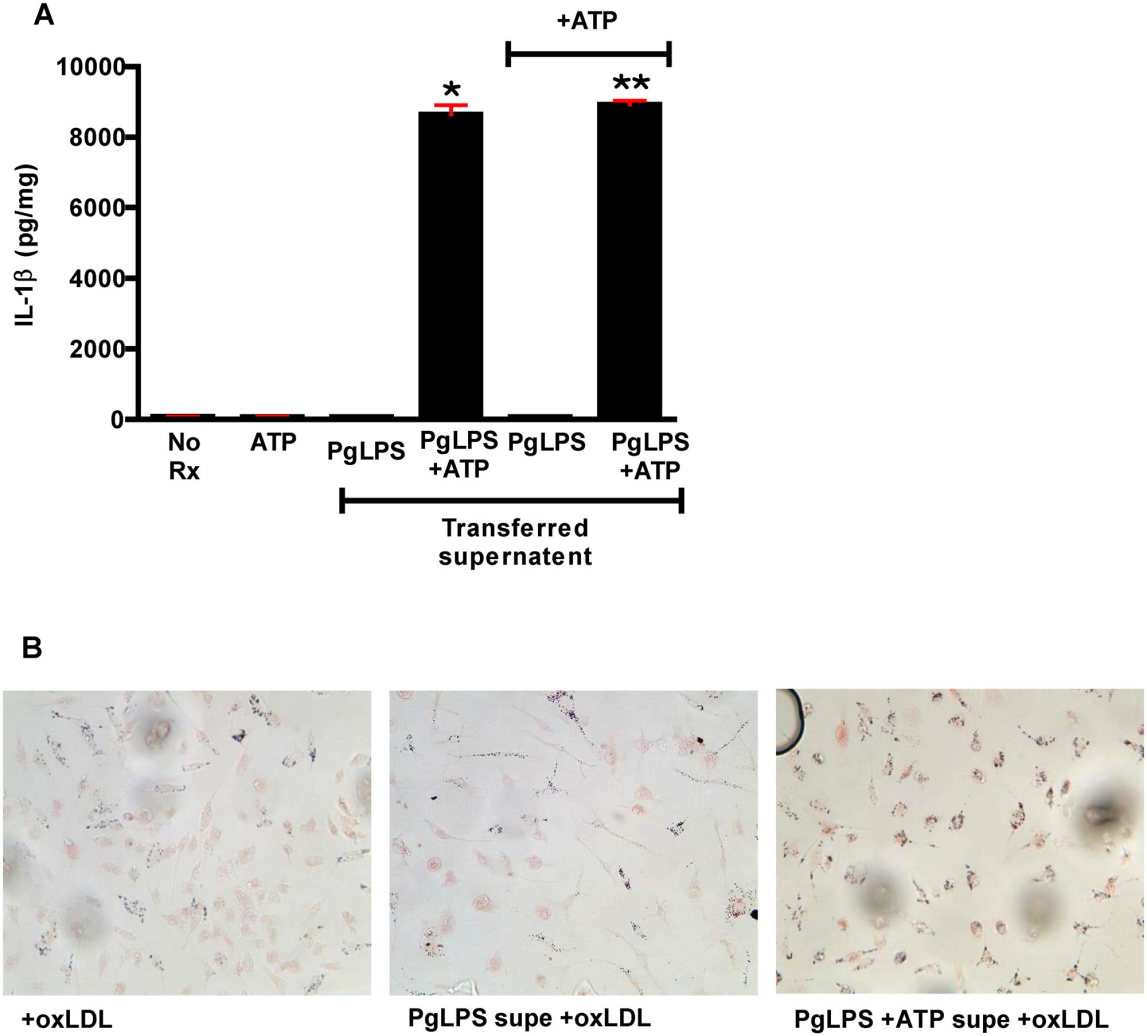
IL1β promotes macrophage IL1β generation and foam cell formation in the absence of new PgLPS. **A.** Primary macrophages were pre-treated with media alone (no Rx), ATP, or supernatents from cells treated with PgLPS alone or PgLPS+ATP. The IL1β present in the supernatents of cells treated with PgLPS+ATP led to IL1β generation by naïve macrophages without exposure to PgLPS. De novo addition of ATP ruled out passage of PgLPS in supernatents. One way ANOVA, p< 0.0001. Bonferroni’s Multiple Comparison Test, p<0.05. *vs all groups except (PgLPS+ATP)+ATP. ** vs all groups except (PgLPS+ATP). Experiment was performed in quadruplicate and repeated twice. **B**. Primary macrophages were incubated with oxLDL alone or oxLDL + supernatants from cells incubated with PgLPS alone or PgLPS+ATP, and stained with oil red O (oxLDL @ 25μg/ml in DMEM + 1% FBS for 5 hours). Macrophages incubated with supernatants from cells exposed to PgLPS+ATP showed greater lipid accumulation/foam cell formation in response to added oxLDL. Experiment was performed in triplicate and repeated once.

We next tested whether IL1β itself could potentiate IL1β generation. We incubated WT macrophages with PgLPS with or without ATP and collected the supernatants, which we then added, without additional PgLPS, to naïve cells (Fig 14A). We found that if no ATP had been added to the cells providing the supernatant, no IL1β was generated by the naive cells (3rd bar). However, when naïve macrophages were treated with supernatants from cells incubated with ATP and PgLPS, there was robust IL1β generation (4th bar). As shown by the last two bars, addition of ATP along with the supernatants had no effect on IL1β generation, ruling out the possibility that we had transferred PgLPS in the supernatants. These data show that macrophage treatment with IL1β stimulates release of new IL1β. Others have shown that IL1 receptor signaling, independent of caspase1 activation, can lead to IL1β production [62,68].

Phenotypically, addition of supernatants from PgLPS + ATP treated macrophages to naïve macrophages in the presence of oxLDL led to increased foam cell formation compared with macrophages incubated with oxLDL alone, or oxLDL and supernatants from cells treated with PgLPS without ATP (Fig 14B). Thus, IL1βnot only promotes an increased inflammatory response, but an increased atherogenic response.

### PgLPS-associated lethality (pyroptosis) is decreased in the absence of CD36/SR-B2 and in the presence of oxLDL

**Fig 15:**
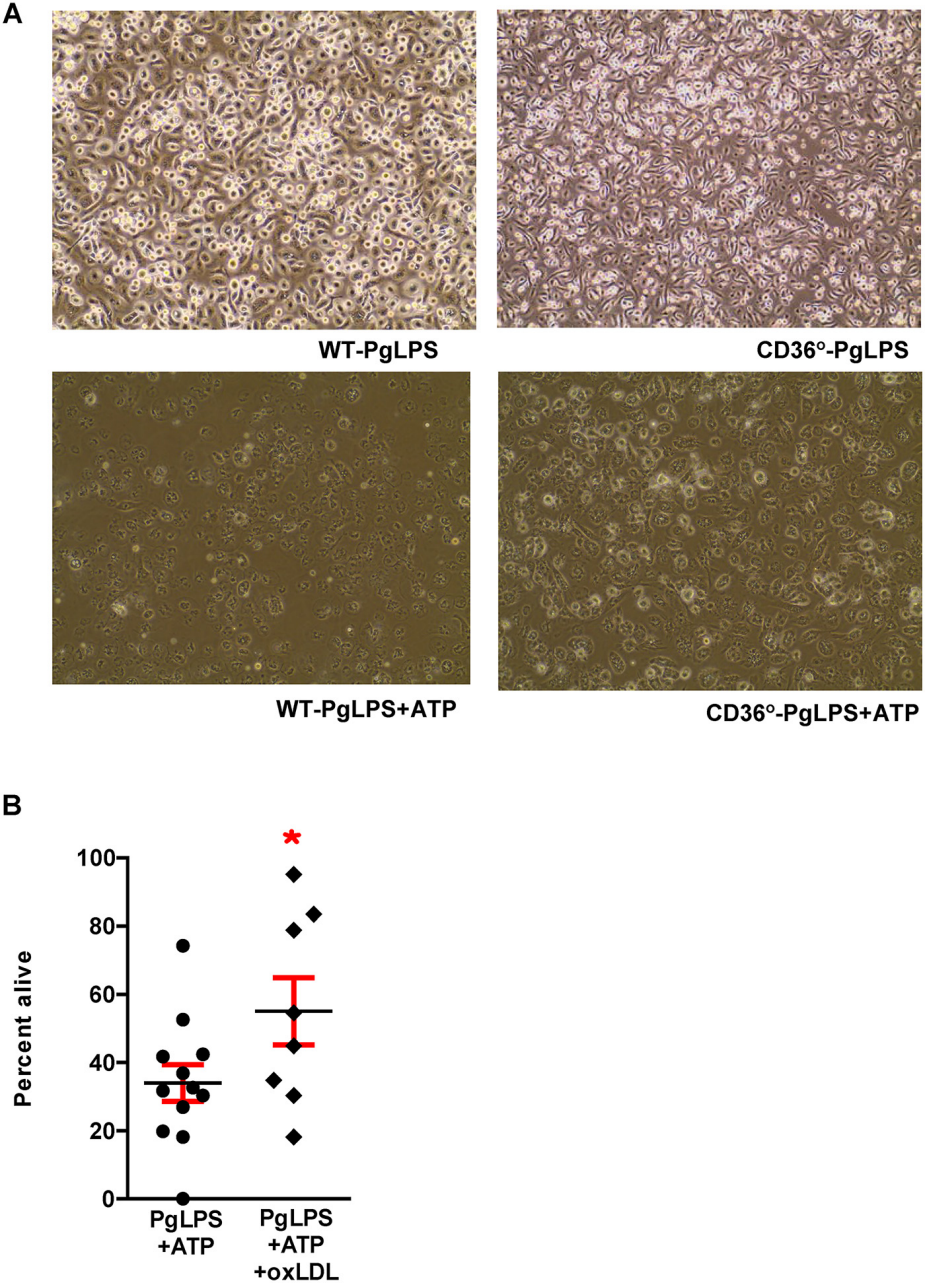
Pyroptosis is reduced in *Cd36*° macrophages and by addition of oxLDL. **A.** WT and *Cd36* **°** macrophages treated with PgLPS showed similar viability, but after addition of 5mM ATP (5hrs), significantly fewer *Cd36*° macrophages died. Experiment was performed in triplicate and repeated twice. **B**. Comparison of ratio of cell protein remaining after treatment to no treatment controls reveals that oxLDL protects against pyroptosis. Two tailed t test, *p<0.05. Experiment was performed in triplicate and repeated once.

**Fig 16:**
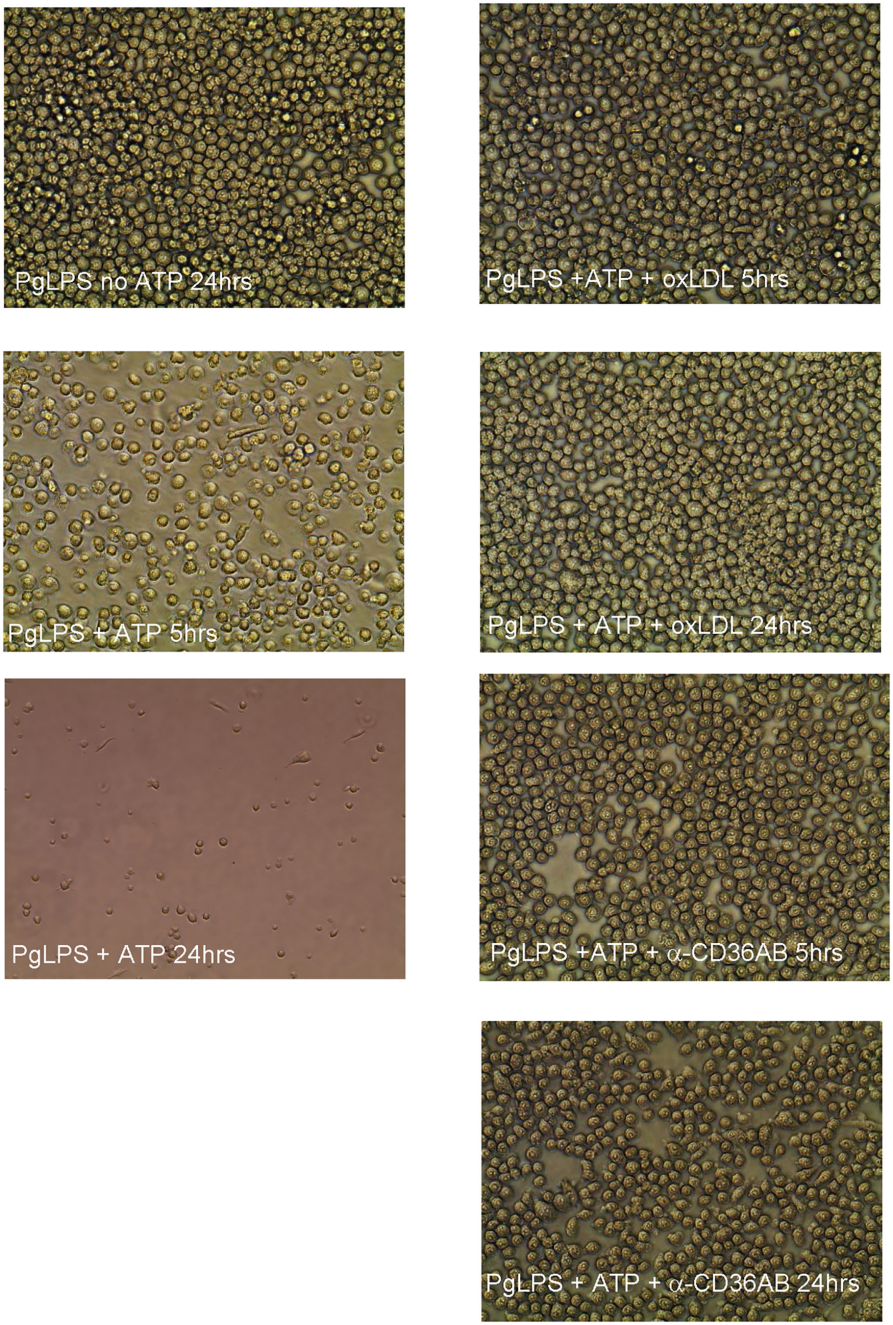
Monoclonal antibody to CD36/SR-B2 and oxLDL inhibit cell death in RAW macrophages. PgLPS, without the addition of ATP, has no effect on cell morphology/cell number, even after 24 hours. Within 5 hours of ATP exposure, membrane blebbing, chromatin condensation and cytoplasmic expulsion are evident. By 24 hours, marked cell death leads to few remaining adherent cells. In contrast, pre-treatment with monoclonal antibody to CD36/SR-B2 (α-CD36 AB) or oxLDL protects against these morphological changes and cell death.

Absence of CD36/SR-B2 led to increased cell survival after exposure to PgLPS + ATP (Fig 15A). Similarly, addition of oxLDL also protected macrophages against PgLPS-mediated lethality (Fig 15B). We observed that RAW cells exposed to PgLPS + ATP had morphological changes associated with cell death, including membrane blebbing and chromatin condensation, occurring within 5 hours (Fig 16). By 24 hours, few cells remained alive and adherent. In support of studies in macrophages from *Cd36*° mice, pre-treatment with monoclonal antibody to CD36 (α-CD36 AB) protected against these affects, as did pre-treatment with oxLDL (Fig 16).

## Discussion

An association between PD and cardiovascular disease has been reiterated recently, despite the absence of a specific mechanism underlying the link between the diseases [10]. We hypothesized that infection in the oral cavity would have a systemic pro-inflammatory effect, through increasing overall oxidative stress and levels of CD36/SR-B2 ligands (oxLDL/KOdiA-PC), and this would lead to greater CD36/SR-B2-dependent lesion burden. Using the *Ldlr*° atherosclerosis mouse model, we found that Pg infection led to increased atherosclerosis lesion burden that was not associated with specific changes in plasma levels of cholesterol, triacylglycerol, non-esterified fatty acids, VLDL/LDL, HDL, or weight. Pg infection did increase cytokine levels of IL6 and IFNγ, both of which have been shown to be increased in mouse models of PD and in human disease. In contrast, there was no increased lesion burden in *Cd36*°*/Ldlr*° mice, despite having similar levels of bone loss, IL6 and IFNγ. Absence of CD36/SR-B2 had no effect on Pg-macrophage association and uptake.

To better understand how Pg infection promoted atherosclerosis, we used isolated primary macrophages to probe mechanism. We found that modified forms of LDL, oxLDL or KOdiA-PC in combination with PgLPS, increased foam cell formation confirming previous observations [69–71]. In addition macrophages from WD fed mice showed enhanced foam cell formation in the presence of PgLPS compared with macrophages from NC fed mice.

IL1β, a pivotal cytokine in atherogenesis, is translated as a pro-protein that is cleaved after activation of caspase1 and released during pyroptosis [72]. The pathway to increased IL1β expression is TLR-dependent and PgLPS is a known TLR ligand. IL1β has long been associated with PD [73,74], and a recent paper using an intravenous Pg infection model showed a significant increase in IL1β secreting cells in the spleen[75]. Because CD36/SR-B2 and TLR may interact, we investigated IL1β release as a marker of TLR activation in macrophages from WT and *Cd36*° mice and found that CD36/SR-B2 is essential for maximal generation of IL1β in response to PgLPS. This was a surprising finding because CD36/SR-B2 was previously thought to interact only with cell wall components of gram positive bacteria [35], but confirms the work of Triantafilou *et al*. [76]. In a similar fashion, we found that TLR2 plays a non-redundant role in IL1β generation in response to PgLPS. TLR2 may form heterodimers with TLR1 and TLR6 [77]. Triantafilou *et al*. showed that TLR2/1 heterodimers mediated PgLPS interaction and uptake, and this also involved CD36/SR-B2 [76]. They did not measure IL1β as an outcome, however.

Using a series of inhibitors, we followed the pathway of IL1β generation. Our data strongly implicate NFκB signaling and the NALP3 inflammasome. From this, we constructed the following working model: in the oral cavity, Pg infection in the presence or absence of CD36/SR-B2 leads to PD with similar bone loss presumably because the cells involved at the forefront of PD infection, neutrophils and T-cells, do not express CD36/SR-B2. However, macrophage interaction with PgLPS leads to CD36/SR-B2 and TLR dependent IL1β generation. The generation of IL1β in the oral cavity as a result of Pg infection is well supported by previous studies [73,74].

There is controversy as to whether Pg/PgLPS is found in atherosclerotic lesions. We did not find evidence of bacteria in aortic arch lesions or blood, but we did not do extensive sampling, and this does not rule out a “hit and run” scenario, where the bacteria are present early in lesion development/oral infection, or the presence of PgLPS. A recent paper, using a more chronic infection protocol (oral lavage for 4 consecutive days every 3^rd^ week for 12 or 24 weeks) showed the presence of viable bacteria in atherosclerotic lesions in *Apoe*° mice [51]. Our study paradigm differed, which may explain the disparity in results. In light of this controversial issue, we considered vascular effects both in the presence and absence of PgLPS. We found that IL1β generation was substantially increased in PgLPS-exposed macrophages from WD fed mice compared to those from NC fed mice, suggesting that diet affected inflammatory responses. Transfer of supernatants from macrophages treated with PgLPS + ATP to naïve macrophages led to enhancement of their IL1β response and increased foam cell formation. These data suggest that IL1β release in the oral cavity can strongly potentiate responses in the vasculature, and this may be one mechanism by which PD increases atherosclerosis lesion burden.

We next considered the scenario of naïve macrophages encountering both PgLPS and diet derived oxLDL/KOdiA-PC in the vessel wall. We found that oxLDL/KOdiA-PC strongly inhibited IL1β generation in macrophages from WT and *Tlr2*° but not *Cd36*° mice. Several reports have shown that oxLDL alone can prime/activate inflammasomes [78–80]. However, even at very high oxLDL concentration (200 ug/ml) macrophage secreted IL1β was significantly lower compared with what we observed, and is more consistent with the effects of crystals that may have formed in the oxLDL preparation[80]. Our data are in line with those of Bochkov *et al*. [81], and Walton *et al*. [82], who showed that oxPAPC/oxLDL had inhibitory effects on TLR signaling. Both groups primarily were studying *E*.*coli* LPS, a TLR4 ligand, but Walton *et al*. showed that the effect of oxPAPC did extend to TLR2 ligands. They also showed that KOdiA-PC is one of the most potent inhibitory lipids in oxPAPC.

**Fig 17:**
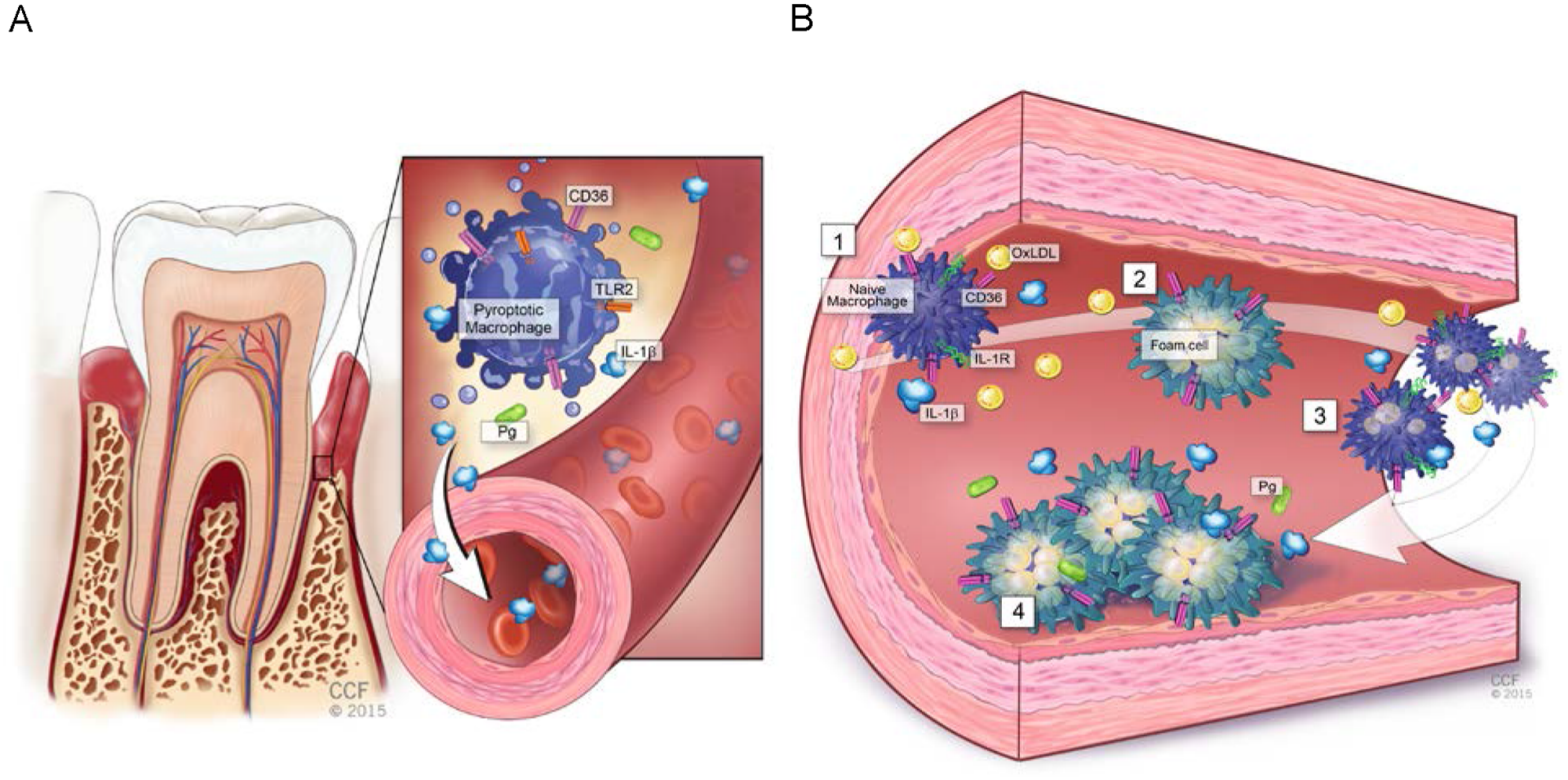
Summary. **A**. Activation of the inflammasome by periodontal disease bacteria (*Porphyromonas gingivalis*, Pg) in the oral cavity is mediated by CD36/SR-B2 and TLR2 and leads to systemic release of pro-atherosclerotic IL1β while inducing macrophage pyroptosis. **B**. Systemic IL1β activates naïve (to Pg) vascular macrophages to secrete IL1β, and promotes CD36-mediated uptake of oxLDL (2) and enhanced foam cell formation (3). Although it is controversial as to whether Pg/PgLPS is found in the vasculature, the presence of oxLDL would inhibit Pg/PgLPS inflammasome activation and pyroptosis, allowing the development of greater atherosclerotic plaque (4).

In addition to inhibition of IL1βgeneration, we found that oxLDL also inhibited LPS-mediated macrophage cell death (pyroptosis). Our data suggest a model (Fig 17) in which Pg infection in the oral cavity leads to robust systemic IL1β that then itself can stimulate IL1β generation by macrophages in the vasculature, and enhance local pro-atherogenic processes. But more potentially compelling, however, is the finding that if both Pg/PgLPS and oxLDL are present in the vasculature, they promote increased foam cell formation, and paradoxically macrophage survival and in this way contribute mechanistically to increased lesion burden as a result of PD.

Building on this emerging concept of inflammatory disease exacerbation due to Pg-mediated PD, another recent study found that chronic oral infection with Pg prior to induction of arthritis in mice led to enhanced immune activation, inflammatory cytokine production, and disease progression [83]. Also in line with our data is a recent report describing decreased atherosclerosis in Pg challenged *LDLR*° mice after immunization with malondialdehyde-modified LDL, supporting the concept that both Pg and modified LDL play a role in increased atherosclerosis [84].

Together, these results show a role for CD36/SR-B2 at multiple points in Pg mediated enhanced atherosclerosis, and support the hypothesis that TLR-CD36/SR-B2 mediated IL1β generation, leading to increased foam cell formation, may be essential to the enhancement in atherosclerosis lesion observed. The importance of ligands generated as a result of hyperlipidemia also suggests that patients with PD should be advised about the apparent increased risk that oral infection adds to high fat diets in the development of cardiovascular disease.

## Supporting Information

S1 File Reagents. (DOCX)
