# Correction: CD36/SR-B2-TLR2 Dependent Pathways Enhance *Porphyromonas gingivalis* Mediated Atherosclerosis in the *Ldlr* KO Mouse Model

**DOI:** 10.1371/journal.pone.0130245

**Published:** 2015-06-10

**Authors:** The PLOS ONE Staff

There is an error in the fourth sentence of the Abstract. The correct sentence is: This increase was entirely CD36/SR-B2-dependent, as there was no significant change in lesion burden between infected and uninfected *Cd36* ^*o*^ */Ldlr* ^*o*^ mice. The publisher apologizes for this error.

## References

[1] Brown PM, Kennedy DJ, Morton RE, Febbraio M (2015) CD36/SR-B2-TLR2 Dependent Pathways Enhance *Porphyromonas gingivalis* Mediated Atherosclerosis in the *Ldlr* KO Mouse Model. PLoS ONE 10(5): e0125126 doi: 10.1371/journal.pone.0125126 25938460 10.1371/journal.pone.0125126 PMC4418723

