# Supplementary material for: CD36/SR-B2-TLR2 Dependent Pathways Enhance Porphyromonas gingivalis Mediated Atherosclerosis in the Ldlr KO Mouse Model
Source: PLoS One. 2015 May 4;10(5):e0125126. doi: 10.1371/journal.pone.0125126 (PMC4418723; doi:10.1371/journal.pone.0125126)
Supplement: S1 File — (DOCX) [file pone.0125126.s001.docx]

**Reagents**

Dulbecco’s Modified Eagle’s Medium (DMEM), phosphate buffered saline (PBS), penicillin (10,000 units)/streptomycin (10mg/ml) were from the Lerner Research Institute Media Core, Cleveland Clinic. Fetal bovine serum was from Atlanta Biologicals (S11150). Ready PCR Mix 2x (N806) was from Ameresco. Biotech grade agarose (CA3510-8) and Choice-Taq Blue DNA polymerase (CB4060-1) were from Denville Scientific. PCR primers were synthesized by Integrated DNA Technologies. Crystalized phenol (ACS grade, A92-500), Formalde-Fresh (SF94-4), chloroform (ACS grade, C298SK-4), ethanol (200 proof, Decon Laboratories, 04-355-222) and miscellaneous laboratory chemicals were from Fisher Scientific. Oil red O (19056) and paraformaldehyde (15710) were from EM Sciences. Proteinase K (EO0491) and DNA molecular weight markers (SM0323) were from Fermentas Life Sciences. Porphyromonas gingivalis (Pg, strain 33277, 9008) was from Gibson Laboratories, Inc. PgLPS (tlrl-pglps), z-VAD-FMK (tlrl-vad), monoclonal antibody to mouse TLR2 (mab-mtlr2), ATP (tlrl-atp), RAW-Blue cells (raw-sp) and QUANTI-Blue (rep-qb1) were from InvivoGen. The Database of Cross-Contaminated or Misidentified Cell Lines maintained by the International Cell Line Authentication Committee (ICLAC) (version 7.2, released 10 October 2014) was consulted and RAW Blue cells were not found. Polyclonal antibody to TLR2 (121801) was from BioLegend. z-YVAD-FMK (1141-5) was from BioVision. N-[1-[[(Cyanoamino)(5-quinolinylami¬no)methylene]amino]-2,2-dimethylpropyl]-3,4-dimeth-oxybenzeneacetamide (A740003, 3701) was from Tocris Bioscience. 1-(Palmitoyl)-2-(5-keto-6-octene-dioyl) phosphatidylcholine (KOdiA-PC, 62945), and caffeic acid phenethyl ester (CAPE, 70750) were from Cayman Chemical. (2R,4R)-4-aminopyrrolidine-2, 4-dicarboxylate (APDC, sc-202408), L-NG-nitroarginine methyl ester (L-NAME-HCl, sc-200333) and 3,4,5-trimethoxybenzoic acid 8-(diethylamino)octyl ester (TMB8, sc-3522) were from Santa Cruz Biotechnology, Inc. Diphenyleneiodonium chloride (DPI, D2926), resveratrol (R50-10), methylene blue (M9140), carboxymethyl cellulose sodium salt (medium viscosity, C4888) and polymyxin B agarose (P1411) were from Sigma-Aldrich. Thrombospondin-1 from human platelets was from American Research Products, Inc. (12-7616). Native LDL was prepared by sequential centrifugation as previously described.[40] OxLDL was prepared as previously described.[41] Syto 17 (S-7579) and Alex Fluor 488 goat anti-mouse secondary antibody (A10667) were from Molecular Probes-Invitrogen. Monoclonal anti-CD36/SR-B2 antibody (IgA clone, 552544), isotype control (mouse IgA Kappa, 553476), Difco Brewer’s thioglycollate medium (211716) and Schaedler broth with Vitamin K1 (221541) were from BD Pharmingen. FcR blocking reagent (130-092-575) was from Miltenyi Biotech. DC Protein Assay (500-0116), EDTA (161-0729) and Tris (161-0716) were from Bio-Rad.
